# A Novel *in vivo* Anti-amnesic Agent, Specially Designed to Express Both Acetylcholinesterase (AChE) Inhibitory, Serotonergic Subtype 4 Receptor (5-HT_4_R) Agonist and Serotonergic Subtype 6 Receptor (5-HT_6_R) Inverse Agonist Activities, With a Potential Interest Against Alzheimer’s Disease

**DOI:** 10.3389/fnagi.2019.00148

**Published:** 2019-06-19

**Authors:** Bérénice Hatat, Samir Yahiaoui, Cédric Lecoutey, Audrey Davis, Thomas Freret, Michel Boulouard, Sylvie Claeysen, Christophe Rochais, Patrick Dallemagne

**Affiliations:** ^1^Normandie Université, UNICAEN, Centre d’Etudes et de Recherche sur le Médicament de Normandie (CERMN), Caen, France; ^2^IGF, University of Montpellier, CNRS, INSERM, Montpellier, France; ^3^Normandie Université, UNICAEN, INSERM, U1075, GIP CYCERON, COMETE, Caen, France

**Keywords:** Alzheimer’s disease, acetylcholinesterase, 5-HT_4_ receptors, 5-HT_6_ receptors, MTDL

## Abstract

This work describes the conception, synthesis, *in vitro* and *in vivo* biological evaluation of novel Multi-Target Directed Ligands (MTDL) able to both activate 5-HT_4_ receptors, block 5-HT_6_ receptors and inhibit acetylcholinesterase activity (AChE), in order to exert a synergistic anti-amnesic effect, potentially useful in the treatment of Alzheimer’s disease (AD). Indeed, both activation of 5-HT_4_ and blockage of 5-HT_6_ receptors led to an enhanced acetylcholine release, suggesting it could lead to efficiently restoring the cholinergic neurotransmission deficit observed in AD. Furthermore, 5-HT_4_ receptor agonists are able to promote the non-amyloidogenic cleavage of the amyloid precursor protein (APP) and to favor the production of the neurotrophic protein sAPPα. Finally, we identified a pleiotropic compound, [1-(4-amino-5-chloro-2-methoxyphenyl)-3-(1-(3-methylbenzyl)piperidin-4-yl)propan-1-one fumaric acid salt (**10**)], which displayed *in vivo* an anti-amnesic effect in a model of scopolamine-induced deficit of working memory at a dose of 0.3 mg/kg.

**Graphical Abstract:**
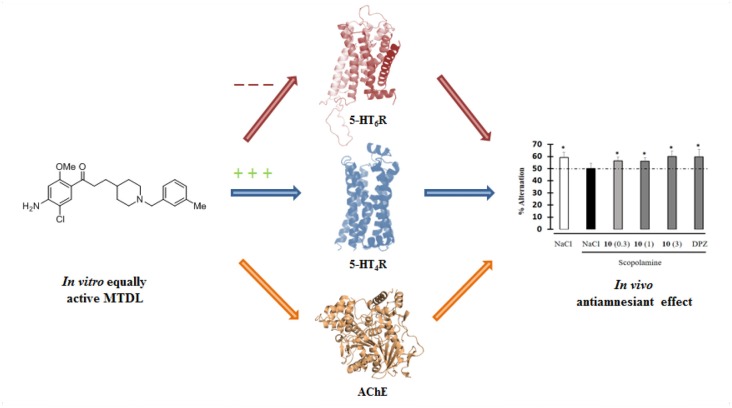
Design of a MTDL able *in vitro* to both inactivate 5-HT6R and AChE and to activate 5-HT4R and finally displaying *in vivo* antiamnesiant effect.

## Introduction

The pathogenesis of Alzheimer’s Disease (AD) is complex and related to the abnormality and dysfunction of multi-systems. Thus, to be potentially more effective, a treatment might consider more than a single target such as acetylcholinesterase (AChE) the main focus of marketed AD drugs.

**Figure 1:**
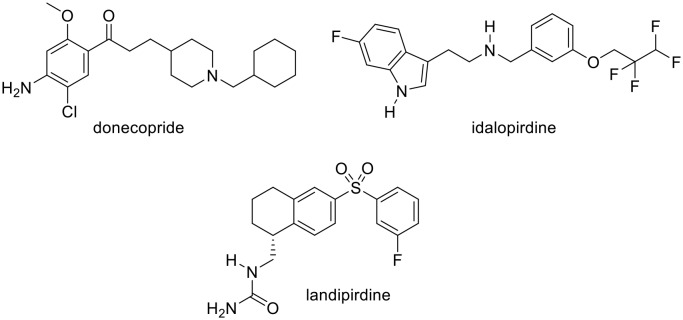
Structure of donecopride, idalopirdine and landipirdine.

Within this framework, the design of some pleiotropic ligands known as Multi-Target Directed Ligands (MTDL; Cavalli et al., 2008) appears as a promising approach to tackle the complex origin of the disease as demonstrated recently for several G Protein-Coupled Receptors (GPCRs) and enzymes (Dolles and Decker, 2017; Dolles et al., 2018). We recently described compound **1** (donecopride), which is currently a novel preclinical drug candidate exhibiting both an *in vitro* dual-binding site AChE inhibitory activity and a serotonergic subtype 4 receptor (5-HT_4_R) agonist effect leading to *in vivo* procognitive and anti-amnesic effects in mice (Figure 1; Lecoutey et al., 2014; Rochais et al., 2015). Indeed, 5-HT_4_R agonists are able to promote the “non-amyloidogenic” cleavage of the amyloid precursor protein (APP) by α-secretase, inducing the decrease in amyloid-β peptide (Aβ) production in primary neurons (Lezoualc’h, 2007; Russo et al., 2009), the release of soluble and neuroprotective sAPPα protein (Cho and Hu, 2007), and the *in vivo* improvement of memory in rodents (Lelong et al., 2003; Nirogi et al., 2018).

5-HT_4_R is a GPCR. Interestingly, another serotonin GPCR, the serotonergic subtype 6 receptor (5-HT_6_R) appears as a valuable target to treat cognitive impairments in the field of neurodegenerative disorders, notably AD (Karila et al., 2015). In fact, its blockade confers to 5-HT_6_R antagonists procognitive effects (Benhamú et al., 2014). Among these 5-HT_6_R antagonists, **2** (idalopirdine) was studied in phase 3 of clinical trials (Wilkinson et al., 2014), and **3** (landipirdine), a dual antagonist of the 5-HT_6_ and 5-HT_2A_ receptors is currently under investigation in the field of Parkinson Disease (Figure 1; Ellis and Fell, 2017).

**Figure 2:**
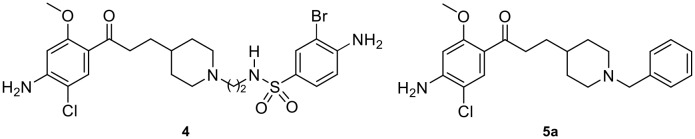
Structure of compounds **4** and **5a**.

The procognitive activity of 5-HT_6_R antagonists is probably mediated by modulation of neurotransmitters’ release. Indeed, 5-HT_4_R activation enhances the liberation of acetylcholine (Kilbinger and Wolf, 1992; Consolo et al., 1994), dopamine (Steward et al., 1996; Lucas et al., 2001) and serotonin (Ge and Barnes, 1996), while blockade of 5-HT_6_R enhances the liberation of acetylcholine (Shirazi-Southall et al., 2002; Riemer et al., 2003; Hirst et al., 2006; Marcos et al., 2006; Zhang et al., 2007) and glutamate (Dawson et al., 2000, 2001). Consequently, 5-HT_6_R antagonists can improve cognition since they limit the activation of the mTOR pathway (de Bruin and Kruse, 2015). The acute administration of 5-HT_4_R agonists or 5-HT_6_R antagonists leads to procognitive effects. However, recently we demonstrated that chronic 5-HT_4_R activation or chronic 5-HT_6_R blockade, also improved memory performances in object recognition test in mice (Quiedeville et al., 2015). These ligands are active at lower doses than those needed for an acute effect and furthermore, seem to be devoid of side effects in these conditions. We thus considered that co-modulation of these two receptors could represent a valuable strategy against memory deficits in AD (Claeysen et al., 2015; Lalut et al., 2017). Therefore, we recently reported the synthesis and the *in vivo* procognitive effect displayed by the dual compound **4** with *in vitro* 5-HT_4_R agonist and 5-HT_6_R antagonist effects (Figure 2; Yahiaoui et al., 2016). Based on the evidence that 5-HT_4_R, 5-HT_6_R and AChE are all valuable therapeutic targets in AD treatment, the present work aimed at designing, starting from our dual compound **4** and the benzyl analog of donecopride **5a** (Rochais et al., 2015), novel MTDLs associating 5-HT_4_R agonist, 5-HT_6_R antagonist and AChE inhibitory balanced activities (Figure 2).

## Experimental Section

### Chemistry

#### General Materials and Methods

All chemical reagents and solvents were purchased from commercial sources and used without further purification. Melting points were determined on a STUART SMP50 melting point apparatus. ^1^H, ^13^C and ^19^F NMR spectra were recorded on a BRUKER AVANCE III 400MHz with chemical shifts expressed in parts per million (in chloroform-*d*, methanol-*d*_4_ or DMSO-*d*_6_) downfield from TMS as an internal standard and coupling in Hertz. IR spectra were recorded on a Perkin-Elmer BX FT-IR apparatus using KBr pellets. High resolution mass spectra (HRMS) were obtained by electrospray on a BrukermaXis. The purities of all tested compounds were analyzed by LC−MS, with the purity all being higher than 95%. Analyses were performed using a Waters Alliance 2695 as separating module (column XBridge C18 2.5 μM/4.6 × 50 mM) using the following gradients: A (95%)/B (5%) to A (5%)/B (95%) in 4.00 min. This ratio was held during 1.50 min before return to initial conditions in 0.50 min. Initial conditions were then maintained for 2.00 min (A = H_2_O, B = CH_3_CN; each containing HCOOH: 0.1%). MS were obtained on a SQ detector by positive ESI.

#### Procedures for the Deprotection and *N*-alkylation of *tert*-butyl 4-[3-(4-amino-5-chloro-2-methoxyphenyl)-3-oxo-propyl]piperidine-1-carboxylate (8)

Procedure A: to a solution of compound **8** in DCM (5 mL) was added TFA (1 mL) dropwise. The reaction mixture was stirred at room temperature for 1 h and then concentrated under reduced pressure. Evaporation of the solvent provided a light-yellow oil, which was thereafter dissolved in 1,4-dioxane (30 mL). To the resulting solution, 20 equivalent of K_2_CO_3_ were added and thereafter 1.2 equivalent of commercially available benzylbromide derivative was added. The reaction mixture was then stirred at reflux until the full consumption of the starting material. The mixture was then concentrated *in vacuo*, diluted with water and extracted twice with EtOAc. The combined organic phases were washed with brine, dried over MgSO_4_, filtrated and concentrated under pressure. The crude product was thereafter purified by silica column flash chromatography using the appropriate eluting system.

Procedure B: to a solution of compound **8** in DCM (5 mL/mmol) was added TFA (1 mL/mmol) dropwise. The reaction mixture was stirred at room temperature for 15 min and then concentrated under reduced pressure. Evaporation of the solvent provided a light-yellow oil, which was thereafter dissolved in DMF (10 mL/mmol). To the resulting solution, 10 equivalent of K_2_CO_3_ were added and thereafter 1.2 equivalent of commercially available benzylbromide derivative was added. The reaction mixture was then stirred at 110°C until the full consumption of the starting material. The mixture was then concentrated *in vacuo*, diluted with EtOAc and washed twice with brine. The organic layer was dried over MgSO_4_, filtrated and concentrated under pressure. The crude product was thereafter purified by silica column flash chromatography using the appropriate eluting system.

#### 1-(4-Amino-5-chloro-2-methoxyphenyl)-3-(1-benzyl)piperidin-4-yl)propan-1-one (5a; Rochais et al., 2015)

Following procedure A. Pale yellow oil (23%); ^1^H NMR (400 MHz, CDCl_3_) δ 7.71 (s, 1H), 7.19 (m, 5H), 6.18 (s, 1H), 4.39 (s, 2H), 3.76 (s, 3H), 3.45 (s, 2H), 2.81 (m, 4H), 1.90 (*br* t, *J* = 10.2 Hz, 2H), 1.61 (*br* d, *J* = 9.2 Hz, 2H), 1.52 (q, *J* = 6.9 Hz, 2H), 1.22 (m, 3H); ^13^C NMR (100 MHz, CDCl_3_) δ 199.2, 159.5, 147.7, 137.7, 132.3, 129.5 (2C), 128.2 (2C), 127.1, 118.9, 111.3, 97.5, 63.3, 55.6, 53.7 (2C), 40.8, 35.4, 31.9 (2C), 31.4; HRMS (m/z) calcd for C_22_H_28_ClN_2_O_2_ [M + H]^+^ 387.183382, found 387.183120.

#### 1-(4-Amino-5-chloro-2-methoxyphenyl)-3-(1-(2-chlorobenzyl)piperidin-4-yl)propan-1-one (5b)

Following procedure B. Pale yellow solid (33%); mp 125.4°C; ^1^H NMR (400 MHz, CDCl_3_) δ 7.78 (s, 1H), 7.48 (dd, *J* = 7.7, 1.6 Hz, 1H), 7.32 (dd, *J* = 7.8, 1.4 Hz, 1H), 7.22 (dt, *J* = 7.5, 1.4 Hz, 1H), 7.16 (dt, *J* = 7.7, 1.8 Hz, 1H), 6.25 (s, 1H), 4.47 (s, 2H), 3.83 (s, 3H), 3.59 (s, 2H), 2.92–2.88 (m, 4H), 2.08–2.03 (m, 2H), 1.69–1.67 (m, 2H), 1.62–1.57 (m, 2H), 1.30–1.23 (m, 3H); ^13^C NMR (100 MHz, CDCl_3_) δ 199.3, 159.6, 147.8, 136.5, 134.3, 132.3, 130.8, 129.4, 128.0, 126.7, 119.1, 111.4, 97.7, 59.7, 55.7, 54.1 (2C), 41.1, 35.7, 32.5 (2C), 31.5; MS (m/z) [M + H]^+^ 421.61–423.63; HRMS (m/z) calcd for C_22_H_27_Cl_2_N_2_O_2_ [M + H]^+^ 421.1450, found 421.1462.

#### 1-(4-Amino-5-chloro-2-methoxyphenyl)-3-(1-(2-bromobenzyl)piperidin-4-yl)propan-1-one (5c)

Following procedure B. Pale brown solid (43%); mp 118.1°C; ^1^H NMR (400 MHz, CDCl_3_) δ 7.78 (s, 1H), 7.51 (dd, *J* = 7.9, 1.1 Hz, 1H), 7.48 (dd, *J* = 7.3, 1.5 Hz, 1H), 7.26 (dt, *J* = 7.5, 1.1 Hz, 1H), 7.08 (dt, *J* = 7.8, 1.7 Hz, 1H), 6.25 (s, 1H), 4.49 (s, 2H), 3.82 (s, 3H), 3.56 (s, 2H), 2.92–2.88 (m, 4H), 2.09–2.04 (m, 2H), 1.69–1.67 (m, 2H), 1.62–1.57 (m, 2H), 1.28–1.25 (m, 3H); ^13^C NMR (100 MHz, CDCl_3_) δ 199.3, 159.6, 147.8, 138.2, 132.7, 132.3, 130.8, 128.3, 127.3, 124.7, 119.0, 111.3, 97.6, 62.2, 55.7, 54.1 (2C), 41.1, 35.7, 32.5 (2C), 31.4; MS (m/z) [M + H]^+^ 465.59–467.59–469.59; HRMS (m/z) calcd for C_22_H_27_BrClN_2_O_2_ [M + H]^+^ 465.0944, found 465.0945.

#### 1-(4-Amino-5-chloro-2-methoxyphenyl)-3-(1-(2-fluorobenzyl)piperidin-4-yl)propan-1-one (5d)

Following procedure B. Pale yellow solid (56%); mp 115°C; ^1^H NMR (399.75 MHz, CDCl_3_) δ 7.77 (s, 1H), 7.36 (td, *J* = 7.6, 1.8 Hz, 1H), 7.22 (m, 1H), 7.10 (td, *J* = 7.6, 1.2 Hz, 1H), 7.01 (m, 1H), 6.24 (s, 1H), 4.47 (s, 2H), 3.82 (s, 3H), 3.56 (s, 2H), 2.88 (m, 4H), 2.00 (*br* t, *J* = 10.7 Hz, 2H), 1.67 (*br* d, *J* = 9.3 Hz, 2H), 1.58 (m, 2H), 1.27 (m, 3H); ^13^C NMR (100.53 MHz, CDCl_3_) δ 199.1, 161.2 (d, *J* = 244.5 Hz), 159.5, 147.7, 132.2, 131.7 (d, *J* = 4.6 Hz), 128.6 (d, *J* = 8.0 Hz), 125.0 (d, *J* = 14.7 Hz), 123.8 (d, *J* = 3.4 Hz), 118.9, 115.2 (d, *J* = 22.5 Hz), 111.2, 97.5, 63.3, 55.6 (2C), 53.6 (2C), 40.9, 35.5, 32.3, 31.3, 29.7; ^19^F NMR (376.10 MHz, CDCl_3_) δ −117.75 (s); MS (m/z) [M + H]^+^ 405.56–407.55; HRMS (m/z) calcd for C_22_H_27_ClFN_2_O_2_ [M + H]^+^ 405.1745, found 405.1747.

#### 1-(4-Amino-5-chloro-2-methoxyphenyl)-3-(1-(2-iodobenzyl)piperidin-4-yl)propan-1-one (5e)

Following procedure B. Pale yellow solid (44%); mp 106°C;^1^H NMR (400 MHz, CDCl_3_) δ 7.81 (dd, *J* = 7.8, 1.1 Hz, 1H), 7.78 (s, 1H), 7.44 (d, *J* = 7.4 Hz, 1H), 7.30 (dt, *J* = 7.4, 1.1 Hz, 1H), 6.92 (dt, *J* = 7.6, 1.7 Hz, 1H), 6.25 (s, 1H), 4.48 (s, 2H), 3.83 (s, 3H), 3.50 (s, 2H), 2.92–2.88 (m, 4H), 2.11–2.06 (m, 2H), 1.69–1.67 (m, 2H), 1.62–1.57 (m, 2H), 1.31–1.25 (m, 3H) ^13^C NMR (100 MHz, CDCl_3_) δ 199.2, 159.6, 147.8, 141.0, 139.4, 132.3, 130.4, 128.7, 128.1, 119.0, 111.3, 100.7, 97.7, 67.0, 55.8, 54.0 (2C), 41.1, 35.7, 32.4 (2C), 31.4; MS (m/z) [M + H]^+^ 513.59–515.61; HRMS (m/z) calcd for C_22_H_27_ClIN_2_O_2_ [M + H]^+^ 513.0806, found 513.0824.

#### 1-(4-Amino-5-chloro-2-methoxyphenyl)-3-(1-(2-methylbenzyl)piperidin-4-yl)propan-1-one (5f)

Following procedure B. Yellow-pale solid (45%); mp 119°C; ^1^H NMR (400 MHz, CDCl_3_) δ 7.78 (s, 1H), 7.37 (m, 1H), 7.13 (m, 1H), 6.25 (s, 1H), 4.44 (*br* s, 2H), 3.84 (s, 3H), 3.41 (s, 2H), 2.91–2.83 (m, 4H), 2.35 (s, 3H), 1.95 (*br* t, *J* = 11.6 Hz, 2H), 1.67–1.53 (m, 4H), 1.28–1.18 (m, 3H);^13^C NMR (100 MHz, CDCl_3_) δ 199.3, 159.5, 137.4, 137.1, 132.2, 130.1, 129.7, 126.8, 125.4, 119.0, 111.2, 97.6, 61.1, 55.6, 54.1, 41.0, 35.8, 32.5, 31.4, 19.3; MS (m/z) [M + H]^+^ 401.65–403.64; HRMS (m/z) calcd for C_23_H_30_ClN_2_O_2_ [M + H]^+^ 401.1996, found 401.1994.

#### 1-(4-Amino-5-chloro-2-methoxyphenyl)-3-(1-(2-benzyloxybenzyl)piperidin-4-yl)propan-1-one (5g)

Following procedure B. Yellow oil (16%); ^1^H NMR (400 MHz, CDCl_3_) δ 7.78 (s, 1H), 7.46–7.44 (m, 2H), 7.40–7.37 (m, 3H), 7.32 (m, 1H), 7.21 (dt, *J* = 7.9, 1.6 Hz, 1H), 6.96–6.91 (m, 2H), 6.24 (s, 1H), 5.08 (s, 2H), 4.47 (s, 2H), 3.82 (s, 3H), 3.62 (s, 2H), 2.95–2.87 (m, 4H), 2.05–2.00 (m, 2H), 1.68–1.66 (m, 2H), 1.61–1.56 (m, 2H), 1.31–1.26 (m, 3H); ^13^C NMR (100 MHz, CDCl_3_) δ 199.3, 159.6, 157.1, 147.8, 137.5, 132.3, 130.9, 128.6 (2C), 128.0, 127.8, 127.3 (3C), 120.7, 119.1, 112.0, 111.3, 97.7, 70.2, 56.7, 55.7, 54.0 (2C), 41.1, 35.7, 32.5 (2C), 31.5; MS (m/z) [M + H]^+^ 493.65–495.66; HRMS (m/z) calcd for C_29_H_34_ClN_2_O_3_ [M + H]^+^ 493.2258, found 493.2254.

#### 1-(4-Amino-5-chloro-2-methoxyphenyl)-3-(1-(2-hydroxybenzyl)piperidin-4-yl)propan-1-one (5h)

Following procedure B. Yellow-brown oil (12%); ^1^H NMR (400 MHz, CDCl_3_) δ 7.78 (s, 1H), 7.15 (dt, *J* = 8.0, 1.3 Hz, 1H), 6.95 (d, *J* = 7.3 Hz, 1H), 6.80 (d, *J* = 8.1 Hz, 1H), 6.76 (dt, *J* = 7.4, 1.1 Hz, 1H), 6.25 (s, 1H), 4.45 (s, 2H), 3.84 (s, 3H), 3.68 (s, 2H), 3.00–2.97 (m, 2H), 2.90 (t, *J* = 7.8 Hz, 2H), 2.10–2.07 (m, 2H), 1.76–1.73 (m, 2H), 1.63–1.58 (m, 2H), 1.33–1.25 (m, 4H); ^13^C NMR (100 MHz, CDCl_3_) δ 198.9, 159.6, 158.3, 147.8, 132.4, 128.7, 128.6, 122.0, 119.1, 119.0, 116.2, 109.9, 97.7, 61.8, 55.8, 53.4 (2C), 40.8, 35.3, 32.2 (2C), 31.1; MS (m/z) [M + H]^+^ 403.60–405.58; HRMS (m/z) calcd for C_22_H_28_ClN_2_O_3_ [M + H]^+^ 403.1788, found 403.1788.

#### 1-(4-Amino-5-chloro-2-methoxyphenyl)-3-(1-(3-bromobenzyl)piperidin-4-yl)propan-1-one (5i)

Following procedure A. Yellow-pale oil (34%); ^1^H NMR (400 MHz, CDCl_3_) δ 7.78 (s, 1H), 7.47 (*br* t, *J* = 1.6 Hz, 1H), 7.36 (*br* dt, *J* = 1.6, 7.6 Hz, 1H), 7.23 (*br* dt, *J* = 1.6, 7.6 Hz, 1H), 7.16 (t, *J* = 7.6 Hz, 1H), 6.25 (s, 1H), 4.44 (s, 2H), 3.84 (s, 3H), 3.44 (s, 2H), 2.89 (*br* t, *J* = 7.8 Hz, 2H), 2.84 (*br* d, *J* = 10.8 Hz, 2H), 1.93 (*br* t, *J* = 11.0 Hz, 2H), 1.74–1.64 (m, 2H), 1.59 (*br* q, *J* = 6.9 Hz, 2H), 1.34–1.19 (m, 3H); ^13^C NMR (100 MHz, CDCl_3_) δ 199.2, 159.6, 147.8, 141.3, 132.4, 132.1, 130.1, 129.8, 127.9, 122.5, 119.2, 111.4, 97.7, 63.0, 55.8, 54.0, 41.0, 35.7, 32.4, 31.4; HRMS (*m*/*z*) calcd for C_22_H_27_BrClN_2_O_2_ [M + H]^+^ 465.093894, found 465.093548.

#### 1-(4-Amino-5-chloro-2-methoxyphenyl)-3-(1-(3-fluorobenzyl)piperidin-4-yl)propan-1-one (5j)

Following procedure B. White solid (48%); mp 95°C; ^1^H NMR (400 MHz, CDCl_3_) δ 7.78 (s, 1H), 7.25 (m, 1H), 7.06 (m, 2H), 6.92 (m, 1H), 6.25 (s, 1H), 4.44 (s, 2H), 3.84 (s, 3H), 3.46 (s, 2H), 2.87 (m, 4H), 1.94 (*br* t, *J* = 11.1 Hz, 2H), 1.66 (m, 2H), 1.58 (m, 2H), 1.27 (m, 3H); ^13^C NMR (100 MHz, CDCl_3_) δ 199.1, 162.9 (d, *J* = 243.7 Hz), 147.7, 141.5 (d, *J* = 6.7 Hz), 132.2, 129.5 (d, *J* = 8.1 Hz), 124.6 (d, *J* = 2.9 Hz), 118.9, 115.8 (d, *J* = 21.1 Hz), 113.7 (d, *J* = 20.8 Hz), 111.3, 97.6, 62.9, 55.6, 53.9, 40.9, 35.5, 32.3, 31.3; ^19^F NMR (376.10 MHz, CDCl_3_) δ −113.97 (s); MS (m/z) [M + H]^+^ 405.58–407.57; HRMS (m/z) calcd for C_22_H_27_ClFN_2_O_2_ [M + H]^+^ 405.1745, found 405.1745.

#### 1-(4-Amino-5-chloro-2-methoxyphenyl)-3-(1-(3-methylbenzyl)piperidin-4-yl)propan-1-one (5k)

Following procedure A. Yellow-pale oil (41%); ^1^H NMR (400 MHz, CDCl_3_) δ 7.78 (s, 1H), 7.19 (t, *J* = 7.4 Hz, 1H), 7.13 (*br* s, 1H), 7.09 (*br* d, *J* = 7.6 Hz, 1H), 7.06 (*br* d, *J* = 7.2 Hz, 1H), 6.25 (s, 1H), 4.44 (s, 2H), 3.83 (s, 3H), 3.45 (s, 2H), 2.96–2.80 (m, 4H), 2.34 (s, 3H), 1.93 (*br* t, *J* = 10.4 Hz, 2H), 1.73–1.52 (m, 4H), 1.36–1.17 (m, 3H); ^13^C NMR (100 MHz, CDCl_3_) δ 199.3, 159.6, 147.8, 138.3, 137.9, 132.4, 130.2, 128.1, 127.8, 126.6, 119.2, 111.4, 97.7, 63.7, 55.8, 54.0, 41.0, 35.7, 32.3, 31.5, 21.5; HRMS (*m*/*z*) calcd for C_23_H_30_ClN_2_O_2_ [M + H]^+^ 401.1994, found 401.1992.

#### 1-(4-Amino-5-chloro-2-methoxyphenyl)-3-(1-(3-methoxybenzyl)piperidin-4-yl)propan-1-one (5l)

Following procedure A. Colorless oil (27%); ^1^H NMR (400 MHz, CDCl_3_) δ 7.78 (s, 1H), 7.22 (t, *J* = 8.0 Hz, 1H), 6.93–6.85 (m, 2H), 6.79 (ddd, *J* = 0.8, 2.4, 8.0 Hz, 1H), 6.25 (s, 1H), 4.44 (s, 2H), 3.83 (s, 3H), 3.81 (s, 3H), 3.47 (s, 2H), 2.95–2.81 (m, 4H), 1.94 (*br* t, *J* = 10.2 Hz, 2H), 1.75–1.52 (m, 4H), 1.36–1.17 (m, 3H); ^13^C NMR (100 MHz, CDCl_3_) δ 199.3, 159.7, 159.6, 147.7, 140.2, 132.4, 129.2, 121.8, 119.2, 114.9, 112.5, 111.4, 97.7, 63.5, 55.8, 55.4, 54.0, 41.0, 35.7, 32.4, 31.5; HRMS (*m*/*z*) calcd for C_23_H_30_ClN_2_O_3_ [M + H]^+^ 417.193947, found 417.193907.

#### 1-(4-Amino-5-chloro-2-methoxyphenyl)-3-(1-(3-aminobenzyl)piperidin-4-yl)propan-1-one (5m)

Following procedure A. Hygroscopic solid (36%); ^1^H NMR (400 MHz, CD_3_OD) δ 7.66 (s, 1H), 7.17 (t, *J* = 7.8 Hz, 1H), 6.87–6.70 (m, 3H), 6.69 (s, 2H), 6.44 (s, 1H), 4.11 (s, 2H), 3.85 (s, 3H), 3.43 (*br* d, *J* = 12.4 Hz, 2H), 3.05–2.76 (m, 4H), 1.95 (*br* d, *J* = 14.0 Hz, 2H), 1.73–1.28 (m, 5H).

#### 1-(4-Amino-5-chloro-2-methoxyphenyl)-3-(1-(3-nitrobenzyl)piperidin-4-yl)propan-1-one (5n)

Following procedure A. Yellow oil (80%); ^1^H NMR (400 MHz, CDCl_3_) δ 8.18 (*br* t, *J* = 1.6 Hz, 1H), 8.09 (*br* d, *J* = 8.0 Hz, 1H), 7.78 (s, 1H), 7.66 (*br* d, *J* = 7.6 Hz, 1H), 7.47 (t, *J* = 8.0 Hz, 1H), 6.26 (s, 1H), 4.45 (s, 2H), 3.84 (s, 3H), 3.55 (s, 2H), 2.90 (*br* t, *J* = 7.8 Hz, 2H), 2.83 (*br* d, *J* = 11.6 Hz, 2H), 1.99 (*br* t, *J* = 10.2 Hz, 2H), 1.78–1.64 (m, 2H), 1.60 (*br* q, *J* = 6.8 Hz, 2H), 1.35–1.19 (m, 3H). ^13^C NMR (100 MHz, CDCl_3_) δ 199.2, 159.6, 148.4, 147.8, 141.4, 135.2, 132.4, 129.2, 123.9, 122.2, 119.1, 114.4, 97.7, 62.6, 55.8, 54.1, 41.0, 35.6, 32.4, 31.4; HRMS (*m*/*z*) calcd for C_22_H_27_ClN_3_O_4_ [M + H]^+^ 432.168460, found 432.168262.

#### 1-(4-Amino-5-chloro-2-methoxyphenyl)-3-(1-(4-fluorobenzyl)piperidin-4-yl)propan-1-one (5o)

Following procedure B. Yellow pale solid (32%); mp 128°C; ^1^H NMR (400 MHz, CDCl_3_) δ 7.78 (s, 1H), 7.26 (m, 2H), 7.06 (m, 2H), 6.98 (m, 2H), 6.25 (s, 1H), 4.48 (s, 2H), 3.83 (s, 3H), 3.43 (s, 2H), 2.86 (m, 4H), 1.91 (*br* t, *J* = 10.6 Hz, 2H), 1.66 (*br* d, *J* = 8.8 Hz, 2H), 1.58 (m, 2H), 1.25 (m, 3H); ^13^C NMR (100 MHz, CDCl_3_) δ 199.1, 161.9 (d, *J* = 243.0 Hz), 159.5, 147.7, 134.3 (d, *J* = 3.0 Hz), 132.2, 130.7 (d, *J* = 7.9 Hz, 2C), 118.9, 114.9 (d, *J* = 21.1 Hz, 2C), 111.2, 97.5, 62.7, 55.6, 53.8, 40.9, 35.6, 32.3, 31.3 ; ^19^F NMR (376.1 MHz, CDCl_3_) δ −113.97 (s); MS (m/z) [M + H]^+^ 405.62–407.62; HRMS (m/z) calcd for C_22_H_27_ClFN_2_O_2_ [M + H]^+^ 405.1745, found 405.1740.

#### 1-(4-Amino-5-chloro-2-methoxyphenyl)-3-(1-(4-hydroxybenzyl)piperidin-4-yl)propan-1-one (5p)

Following procedure B. Yellow oil (13%); ^1^H NMR (400 MHz, CD_3_OD) δ 7.64 (s, 1H), 7.14–7.12 (m, 2H), 6.74 (m, 2H), 6.43 (s, 1H), 3.84 (s, 3H), 3.44 (s, 2H), 2.93–2.87 (m, 4H), 2.04–1.99 (m, 2H), 1.72–1.70 (m, 2H), 1.57–1.51 (m, 2H), 1.32–1.22 (m, 4H); ^13^C NMR (100 MHz, CD_3_OD) δ 201.0, 161.6, 158.1, 151.7, 132.9, 132.4 (2C), 128.2, 117.9, 116.0 (2C), 111.6, 98.1, 63.7, 56.1, 54.4 (2C), 41.7, 36.6, 32.6 (3C); MS (m/z) [M + H]^+^ 403.65–405.66; HRMS (m/z) calcd for C_22_H_28_ClN_2_O_3_ [M + H]^+^ 403.1788, found 403.1786.

#### 1-(4-Amino-5-chloro-2-methoxyphenyl)-3-(1-(4-methylbenzyl)piperidin-4-yl)propan-1-one (5q)

Following procedure B. White solid (20%); mp 97.0°C; ^1^H NMR (400 MHz, CDCl_3_) δ 7.77 (s, 1H), 7.20 (d, *J* = 7.9 Hz, 2H), 7.11 (d, *J* = 7.9 Hz, 2H), 6.24 (s, 1H), 4.49 (*br* s, 2H), 3.81 (s, 3H), 3.47 (s, 2H), 2.90–2.86 (m, 4H), 2.33 (s, 3H), 1.96–1.91 (m, 2H), 1.68–1.65 (m, 2H), 1.60–1.55 (m, 2H), 1.33–1.23 (m, 3H); ^13^C NMR (100 MHz, CDCl_3_) δ 199.2, 159.6, 147.8, 136.7, 135.0, 132.3, 129.5 (2C), 128.9 (2C), 118.9, 111.3, 97.6, 63.2, 55.7, 53.8, 53.5, 41.0, 35.6, 32.2 (2C), 31.4, 21.2; MS (m/z) [M + H]^+^ 401.66–403.65; HRMS (m/z) calcd for C_23_H_30_ClN_2_O_2_ [M + H]^+^ 401.1996, found 401.1996.

#### *N*-(3-((4-(3-(4-Amino-5-chloro-2-methoxyphenyl)-3-oxopropyl)piperidin-1-yl)methyl)-4-bromophenyl) acetamide (5r)

Following procedure A. Yellow-pale oil (70%); ^1^H NMR (400 MHz, CDCl_3_) δ 7.77 (s, 1H), 7.96 (*br* s, 1H), 7.52 (dd, *J* = 2.4, 8.8 Hz, 1H), 7.47–7.39 (m, 2H), 6.25 (s, 1H), 4.50 (s, 2H), 3.82 (s, 3H), 3.50 (s, 2H), 3.01–2.77 (m, 4H), 2.16 (s, 3H), 2.05 (*br* t, *J* = 10.8 Hz, 2H), 1.72–1.61 (m, 2H), 1.57 (*br* q, *J* = 6.8 Hz, 2H), 1.35–1.18 (m, 3H). ^13^C NMR (100 MHz, CDCl_3_) δ 199.4, 168.7, 159.7, 147.9, 138.8, 137.5, 133.0, 132.3, 121.6, 120.0, 118.9, 118.8, 111.3, 97.7, 62.0, 55.8, 54.1, 41.1, 35.7, 32.5, 31.4, 24.7; HRMS (*m*/*z*) calcd for C_24_H_30_BrClN_3_O_3_ [M + H]^+^ 522.115358, found 522.114977.

#### 1-(4-Amino-5-chloro-2-methoxyphenyl)-3-(1-(2-fluoro-5-nitrobenzyl)piperidin-4-yl)propan-1-one (5s)

Following procedure B. Yellow solid (35%); mp 133.4°C; ^1^H NMR (400 MHz, CDCl_3_) δ 8.36 (dd, *J* = 6.1 Hz, *J* = 2.8 Hz, 1H), 8.13 (ddd, *J* = 8.9, 4.3 Hz, *J* = 3.0 Hz, 1H), 7.78 (s, 1H), 7.15 (t_app_, *J* = 8.8 Hz, 1H), 6.25 (s, 1H), 4.45 (s, 2H), 3.84 (s, 3H), 3.59 (s, 2H), 2.91–2.84 (m, 4H), 2.08–2.02 (m, 2H), 1.70–1.68 (m, 2H), 1.62–1.57 (m, 2H), 1.29–1.25 (m, 3H); ^13^C NMR (100 MHz, CDCl_3_) δ 199.1, 164.8 (d, *J* = 256.4 Hz), 159.6, 147.8, 144.4 (d, *J* = 1.9 Hz), 132.4, 127.8 (d, *J* = 16.2 Hz), 127.2 (d, *J* = 6.9 Hz), 124.6 (d, *J* = 10.2 Hz), 119.1, 116.3 (d, *J* = 25.1 Hz), 111.4, 97.7, 55.8, 55.1, 53.9 (2C), 40.9, 35.4, 32.3 (2C), 31.3; MS (m/z) [M + H]^+^ 450.51–452.50; HRMS (m/z) calcd for C_22_H_26_ClFN_3_O_4_ [M + H]^+^ 450.1596, found 450.1594.

#### 1-(4-Amino-5-chloro-2-methoxyphenyl)-3-(1-(5-amino-2-fluorobenzyl)piperidin-4-yl)propan-1-one (5t)

Following procedure B. Yellow-brown oil (11%); ^1^H NMR (400 MHz, CDCl_3_) δ 7.64 (s, 1H), 6.83 (dd, *J* = 9.5 Hz, *J* = 8.8 Hz, 1H), 6.72 (dd, *J* = 6.2, 2.8 Hz, 1H), 6.65 (ddd, *J* = 8.7, 4.1, 2.8 Hz, 1H), 6.44 (s, 1H), 3.85 (s, 3H), 3.55 (d, *J* = 1.2 Hz, 2H), 2.98–2.95 (m, 2H), 2.91–2.87 (m, 2H), 2.16–2.11 (m, 2H), 1.73–1.71 (m, 2H), 1.59–1.52 (m, 2H), 1.33–1.26 (m, 3H); ^13^C NMR (100 MHz, CDCl_3_) δ 200.1, 161.7, 156.3 (d, *J* = 234.0 Hz), 151.7, 144.8, 132.9, 124.1 (d, *J* = 16.4 Hz), 119.7 (d, *J* = 3.3 Hz), 117.9, 117.3 (d, *J* = 7.7 Hz), 116.4 (d, *J* = 23.8 Hz), 111.6, 98.1, 56.3 (d, *J* = 1.3 Hz), 56.1, 54.4 (2C), 41.6, 36.4, 32.6 (3C); MS (m/z) [M + H]^+^ 420.60–422.60; HRMS (m/z) calcd for C_22_H_28_ClFN_3_O_2_ [M + H]^+^ 420.1854, found 420.1857.

#### 1-(4-Amino-5-chloro-2-methoxyphenyl)-3-(1-(3-methylbenzyl)piperidin-4-yl)propan-1-one fumaric acid salt (10)

To a solution of 85 mg of compound **5k** (0.212 mmol) in 3 mL of iPrOH was added 25 mg of fumaric acid (0.212 mmol, 1 equivalent). The solution was refluxed for 2 h. The mixture was then concentrated *in vacuo*. The residue is triturated in Et_2_O and then filtrated. Sixty milligram of desired compound were obtained as a beige solid (55%). Yellow solid; mp 185.4°C; ^1^H NMR (400 MHz, CD_3_OD) δ 7.56 (s, 1H), 7.28–7.16 (m, 4H), 6.60 (s, 2H), 6.35 (s, 1H), 4.11 (s, 2H), 3.75 (s, 3H), 3.32 (*br* d, *J* = 12.4 Hz, 2H), 2.88–2.81 (m, 4H), 2.28 (s, 3H), 1.86 (*br* d, *J* = 13.9 Hz, 2H), 1.55–1.52 (m, 3H), 1.38–1.32 (m, 2H); ^13^C NMR (100 MHz, CD_3_OD) δ 198.7, 169.8 (2C), 160.3, 150.4, 139.0, 134.8 (2C), 131.5, 130.4, 129.3, 128.8, 127.9, 116.3, 110.3, 96.6, 60.2, 54.7 (2C), 52.1, 48.2, 39.8, 33.1, 30.1, 28.9, 20.0 (2C). HRMS (m/z) calcd for C_23_H_30_ClN_2_O_2_ [M + H]^+^ 401.1994, found 401.1996.

### *In vitro* Biological Studies

#### Pharmacological Characterization of Drugs on Human 5-HT_4_R

For competition studies, 2.5 μg of proteins (5-HT_4(b)_ membrane preparations, HTS110M, Eurofins. Eurofins’ 5-HT_4(b)_ membrane preparations are crude membrane preparations made from their proprietary stable recombinant cell lines to ensure high-level of GPCR surface expression) were incubated in duplicate at 25°C for 60 min in the absence or the presence of 10^−6^ or 10^−8^M of each drug (**9** was used as a reference standard) and 0.2 nM [^3^H]-GR113808 (NET 1152, Perkin Elmer) in 25 mM Tris buffer (pH 7.4, 25°C). At the end of the incubation, homogenates were filtered through Whatman GF/C filters (Alpha Biotech) presoaked with 0.5% polyethylenimine using a Brandel cell harvester. Filters were subsequently washed three times with 1 mL of ice-cold 25 mM Tris buffer (pH 7.4, 4°C). Non-specific binding was evaluated in parallel in the presence of 30 μM serotonin.

For some of these compounds, affinity constants were calculated from five-point inhibition curves using the GraphPad Prism 6 software and expressed as Ki ± SD.

#### Pharmacological Characterization of Drugs on Human 5-HT_6_R

Drugs were evaluated through their possibility to compete for the binding of [^3^H]-LSD on membranes of HEK-293 cells transiently expressing the human 5-HT_6_ receptors (ref. RBHS6M, Perkin Elmer). In brief, 4 μg of proteins were incubated at 37°C for 60 min in duplicate in the absence or the presence of 10^−6^ or 10^−8^M of each drug (**2** was used as a reference standard) and 2.5 nM [^3^H]-LSD (ref. NET638250UC, Perkin Elmer), in 25 mM Tris-HCl buffer (pH 7.4) supplemented with 0.5 mM EDTA. At the end of the incubation, the homogenates were then filtered through Whatman GF/C filters and washed five times with ice-cold 25 mM Tris-HCl buffer. Non-specific binding was evaluated in the presence of 100 μM serotonin. Radioactivity associated to proteins was then quantified and expressed as the percentage of inhibition of the drugs under study.

For some of these compounds, affinity constants were calculated from five-point inhibition curves using the GraphPad Prism 6 software and expressed as K*i* ± SD.

#### Determination of cAMP Production

COS-7 cells were grown in Dulbecco’s modified Eagle medium (DMEM) supplemented with 10% dialyzed fetal calf serum (dFCS) and antibiotics. Cells were transiently transfected by electroporation with plasmids encoding HA-tagged 5-HT_4_R (100 ng/10^6^ cells) or HA-tagged 5-HT_6_R (70 ng/10^6^ cells or 300 ng/10^6^ cells), then seeded in 96-well plates (16,000 cells/well).

5-HT_4_R-induced cAMP production: 24 h after transfection, cells were exposed to the indicated concentrations of 5-HT_4_R ligands in the presence of 0.1 mM of the phosphodiesterase inhibitor RO-20-1724, at 37°C in 100 μL of HBS (20 mM HEPES; 150 mM NaCl; 4.2 mM KCl; 0.9 mM CaCl_2_; 0.5 mM MgCl_2_; 0.1% glucose; 0.1% BSA). After 10 min, cells were then lysed by addition of the same volume of Triton-X100 (0.1%).

Blockade of the 5-HT_6_R-induced cAMP production: 24 h after transfection, cells were exposed to the indicated concentrations of 5-HT_6_R ligands at 37°C in 50 μL of HBS. After 7 min, 5-HT (5.10^−7^M final concentration) in the presence of RO-20-1724 (0.1 mM final concentration), in 50 μL HBS, was added to the wells. After 10 min at 37°C, cells were then lysed by addition of 100 μL of Triton-X100 (0.1%).

Reversion of the basal 5-HT_6_R-induced cAMP production: the 5-HT_6_R expression was augmented by transfection of higher quantities of cDNA (300 ng/10^6^ cells) to increase the basal cAMP production linked to the constitutive activity of the receptor. Twenty-four hours after transfection, cells were exposed to 10^−4^M of 5-HT_6_R ligands in the presence of RO-20-1724 (0.1 mM final concentration), at 37°C in 100 μL of HBS. This experiment is performed in the absence of 5-HT. After 10 min at 37°C, cells were then lysed by addition of 100 μL of Triton-X100 (0.1%).

Quantification of cAMP production was performed by HTRF^®^ by using the cAMP Dynamic kit (Cisbio Bioassays) according to the manufacturer’s instructions.

#### *In vitro* Tests of AChE Biological Activity

Inhibitory capacity of compounds on AChE biological activity was evaluated through the use of the spectrometric method of Ellman et al. (1961). Acetylthiocholine iodide and 5,5-dithiobis-(2-nitrobenzoic) acid (DTNB) were purchased from Sigma Aldrich. AChE from human erythrocytes (buffered aqueous solution, ≥500 units/mg protein (BCA), Sigma Aldrich) was diluted in 20 mM HEPES buffer pH 8, 0.1% Triton X-100 such as to have enzyme solution with 0.25 unit/mL enzyme activity. In the procedure, 100 μL of 0.3 mM DTNB dissolved in phosphate buffer pH 7.4 was added into the 96-well plate followed by 50 μL of test compound solution and 50 μL of enzyme (0.05 U final). After 5 min of preincubation at 25°C, the reaction was then initiated by the injection of 50 μL of 10 mM acetylthiocholine iodide solution. The hydrolysis of acetylthiocholine was monitored by the formation of yellow 5-thio-2-nitrobenzoate anion as the result of the reaction of DTNB with thiocholine, released by the enzymatic hydrolysis of acetylthiocholine, at a wavelength of 412 nm using a 96-well microplate plate reader (BioTek, Synergy 2). Test compounds were dissolved in analytical grade DMSO. Donepezil (DPZ) was used as a reference standard. The rate of absorbance increase at 412 nm was followed every minute for 10 min. Assays were performed with a blank containing all components except acetylthiocholine, in order to account for non-enzymatic reaction. The reaction slopes were compared and the percent inhibition due to the presence of test compounds was calculated by the following expression: 100 − (v_i_/v_0_ × 100) where v_i_ is the rate calculated in the presence of inhibitor and v_0_ is the enzyme activity.

First screening of AChE activity was carried out at a 10^−6^M concentration of compounds under study. For the compounds with significant inhibition (≥50%), IC_50_ values were determined graphically by plotting the % inhibition vs. the logarithm of six inhibitor concentrations in the assay solution using the GraphPad Prism 6 software.

### *In vivo* Biological Studies

#### Animals

Adult male NMRI mice (3 months old, weighing 35–40 g) from Janvier labs (Le Genest-Saint-Isle, France) were used to perform experiments. Mice were housed by ten in standard polycarbonate cages in standard controlled conditions (22 ± 2°C, 55 ± 10% humidity) with a reversed 12 h light/dark cycle (light on at 7 pm). Food and water were available *ad libitum* in the home cage. All experiments were conducted (between 9 am and 3 pm) during the active-dark-phase of the cycle, were approved by the ethics committee of Normandy (CENOMEXA, n°5161) and were in agreement with the European Directives and French law on animal experimentation (personal authorization n° 14–17 for MB and 14–60 for TF).

#### CNS-Activity and Acute Toxicity Test

Behavioral and neurological changes induced by graded doses (1, 10, 100 mg/kg) of the tested derivatives were evaluated in mice, in groups of four, by a standardized observation technique at different times (30 min, 3 and 24 h) after intraperitoneal administration (Morpugo, 1971). Major changes of behavioral data (for example, hypo- or hyperactivity, ataxia, tremors, convulsion, etc…) were noted in comparison to the control group. The approximate DL_50_ of the compounds were also calculated through the quantification of mortality after 24 h. Amphetamine (2 mg/kg), chlorpromazine (10 mg/kg) were used as a stimulant and depressive references, respectively.

#### Locomotor Activity

Locomotion of mice was measured using an actimeter (Imetronic^®^) through infrared detection. Eight individual removable polycarbonate cages (21 cm length, 7 cm wide and 12 cm high), where each mouse was placed, are disposed in the actimeter. Locomotor activity was measured by recording the number of interruption of beams of the red light over a period of 30 min through an attached recording system to the actimeter. Compound **10** was tested at 1, 3 and 10 mg/kg. Amphetamine (2 mg/kg), chlorpromazine (3 mg/kg) were used as a stimulant and depressive references, respectively (Freret et al., 2012).

#### Spatial Working Memory

Anti-amnesic activity of tested compounds was evaluated by reversal of scopolamine (0.5 mg/kg)-induced deficit on spontaneous alternation behavior in the Y maze test (Hooper et al., 1996). The Y maze made of gray plastic consisted of three equally spaced arms (21 cm long, 7 cm wide with walls 15-cm high). The mouse was placed at the end of one of the arms and allowed to move freely through the maze during a 5 min session while the sequence of arm entries was recorded by an observer. An arm entry was scored when all four feet crossed into the arm. An alternation was defined as entries into all three arms on a consecutive occasion. The number of possible alternation is thus the total number of arm entries minus two; the percentage of alternation was calculated as (actual alternation/possible alternation) × 100. Compound **10** was tested at 0.3, 1 and 3 mg/kg. DPZ (1 mg/kg) was tested as a clinical reference.

#### Pharmacological Treatments

Amphetamine, (+)-α-Methylphenethylamine hemisulfate, chlorpromazine hydrochloride, imipramine hydrochloride and scopolamine hydrobromide were purchased from Sigma (France). All those pharmacological compounds were dissolved in NaCl 0.9% as the vehicle were administered IP 30 min before tests, except scopolamine which was subcutaneously administered 20 min before spontaneous alternation test.

### Statistical Analysis

Results were expressed as mean ± SD and were analyzed by one-way analysis of variance (ANOVA), with Statview^®^ software. In case of significance, a SNK (Student-Newman-Keuls) *post hoc* test was realized. Additionally, for the spontaneous alternation test, the percentage of alternation was compared to a theoretical 50% value (random alternation) by an univariate *t*-test. Differences were considered as statistically significant if the *p*-value was strictly under 0.05.

## Results

### Chemistry

**Scheme 1:**
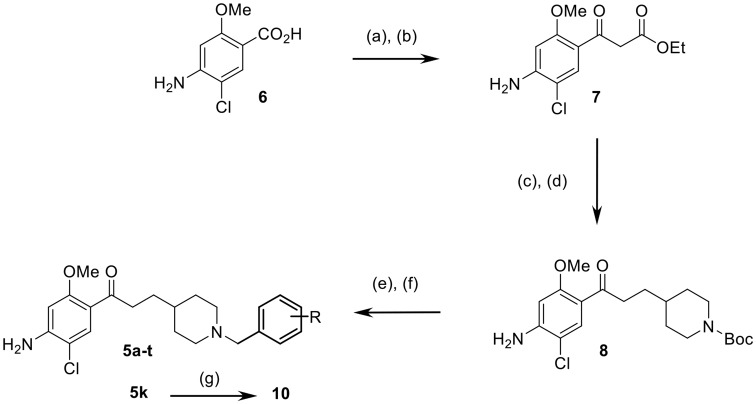
Synthetic pathways for access to compounds **5a–t**, **10**. Conditions and reagents: (a) CDI, THF; (b) KO_2_CCH_2_CO_2_Et, MgCl_2_, THF; (c) *N*-Boc 4-iodomethylpiperidine, K_2_CO_3_, DMF; (d) KOH, EtOH/H_2_O; (e) TFA, 1,4-dioxan or DCM; (f) R-benzyl bromide, K_2_CO_3_, 1,4-dioxane or DMF; (g) fumaric acid, iPrOH.

The targeted compounds **5a–t** were obtained starting from 4-amino-5-chloro-2-methoxybenzoic acid (**6**) as reported for the synthesis of **5a** (Scheme Scheme 1; Rochais et al., 2015). The synthesis of a β-ketoester (**7**) was achieved in 62% yield using the carbonyldiimidazole (CDI) activation of the carboxylic acid group of **6**. A nucleophilic substitution, using *N*-Boc 4-(iodomethyl) piperidine at room temperature to avoid the risk of a double substitution, allowed the installation of the methylpiperidine moiety. The latter was immediately followed by a saponification–decarboxylation sequence using hydroalcoholic potassium hydroxide. The reaction yielded **8** in a 86% yield. Finally, the TFA-*N*-deprotection of **8**, immediately followed by another nucleophilic substitution with various benzyl bromides yielded compounds **5a–t**. The fumaric acid salt **10** was synthesized starting from **5k** and using fumaric acid in iPrOH.

### *In vitro* Results

The biological evaluation of the synthesized compounds as potential inhibitors of human AChE was performed using the Ellman assay (Ellman et al., 1961), as well as their affinity for human 5-HT_6_R and 5-HT_4_R using a radioligand displacement assay (Table 1). In these tests, DPZ was used as a reference AChE inhibitor (AChEI), **2** as a 5-HT_6_R ligand and **9** (RS67333; Eglen et al., 1995) as a 5-HT_4_R ligand.

**Table 1:** (*h*)AChE inhibitory activity, (*h*)5-HT_6_R and (*h*)5-HT_4_R affinity for DPZ, **2**, **9** and compounds **5a–t** (% inhibition at 10^−6^M, 10^−8^M and 10^−8^ M, respectively).

Cmpd	R	(*h*)AChE	(*h*)5-HT_6_R		(*h*)5-HT_4_R	
		%10^−6^M	%10^−6^M	%10^−8^M	%10^−6^M	%10^−8^M
**DPZ**	-	97	-	-	-	-
**2**	-	-	106	88	-	-
**9**	-	-	-	-	100	42
**5a**	H	98	29	0	102	41
**5b**	2-Cl	91	92	27	94	16
**5c**	2-Br	80	65	34	96	17
**5d**	2-F	96	68	21	94	10
**5e**	2-I	37	81	25	96	1
**5f**	2-Me	87	85	24	98	9
**5g**	2-OBn	33	18	0	70	7
**5h**	2-OH	94	24	0	98	10
**5i**	3-Br	44	77	7	94	5
**5j**	3-F	97	59	9	98	12
**5k**	3-Me	89	80	9	98	25
**5l**	3-OMe	35	78	6	94	9
**5m**	3-NH_2_	93	74	31	100	35
**5n**	3-NO_2_	64	51	0	90	3
**5o**	4-F	92	58	13	102	19
**5p**	4-OH	98	24	0	100	23
**5q**	4-Me	87	21	21	100	24
**5r**	2-Br;5-NHCOMe	13	35	7	96	6
**5s**	2-F;5-NO_2_	63	18	5	62	4
**5t**	2-F;5-NH_2_	79	65	3	98	18

**Figure 3:**
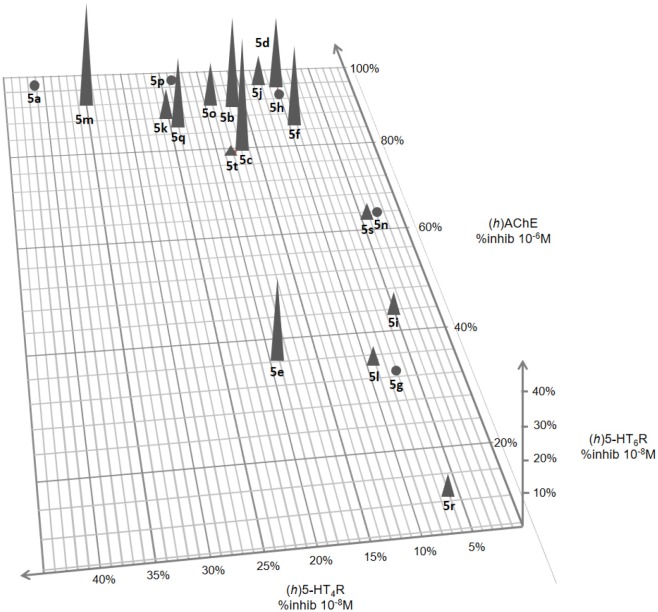
3D representation of (*h*)5-HT_6_R and (*h*)5-HT_4_R affinity and (*h*)AChE inhibitory activity for compounds **5a–t**.

The results of these *in vitro* evaluations are reported in a 3-fold-entry figure (Figure 3).

Among the 20 tested compounds, 12 novel derivatives appear as moderate to potent AChEIs with %inhibition at 10^−6^M ≥ 80%. Their IC_50_ values ranged from 450 (**5q**) to 6 nM (**5p**; Table 2). Some of these AChEI showed an additional affinity towards 5-HT_6_R with %inhibition at 10^−6^M > 70% (**5b, f, k, m**) and K*i* ranging from 1,150 to 110 nM. Three derivatives (**5e, i, l**) displayed affinity for 5-HT_6_R, while being devoid of inhibitory effect towards AChE. Finally, among the four dual compounds, **5k** and **5m** further exhibited an affinity towards 5-HT_4_R with %inhibition at 10^−8^M ≥ 25% and K*i* of 168 and 43 nM respectively. Three other compounds (**5o–q**) displayed good affinity towards 5-HT_4_R but are devoid of such an affinity towards 5-HT_6_R.

**Table 2:** (*h*)AChE inhibitory activity (IC_50_) and (*h*)5-HT_6_R and (*h*)5-HT_4_R affinity (K*i*) for DPZ, **2**, **9**, **5b–f, h–m, o–q, t** and the fumarate **10**.

Cmpd	R	(*h*)AChE	(*h*)5-HT_6_R	(*h*)5-HT_4_R
		IC_50_ (nM) *n* = 2	K*i* (nM) *n* = 3	K*i* (nM) *n* = 3
**DPZ**	-	7 ± 1.5	ND	ND
**2**	-	ND	6.9 ± 1.2	ND
**9**	-	ND	ND	5.1 ± 0.5 (Irving et al., 2010)
**5b**	2-Cl	156 ± 71	110 ± 19	ND
**5c**	2-Br	317 ± 115	ND	ND
**5d**	2-F	101.6 ± 65.4	ND	ND
**5e**	2-I	ND	186 ± 71	ND
**5f**	2-Me	225.2 ± 57.3	270 ± 14	ND
**5h**	2-OH	57.4 ± 9.4	ND	ND
**5i**	3-Br	ND	169 ± 44	ND
**5j**	3-F	91 ± 53.8	ND	ND
**5k**	3-Me	161 ± 43	230 ± 49	168 ± 15
**5l**	3-OMe	ND	265 ± 64	ND
**5m**	3-NH_2_	41 ± 5	1150 ± 559	42.5 ± 2.6
**5o**	4-F	78.4 ± 12.1	ND	ND
**5p**	4-OH	6.0 ± 0.4%	ND	ND
**5q**	4-Me	450.5 ± 89.5	ND	ND
**5t**	2-F;5-NH_2_	152.7 ± 19.9	ND	ND
**10**	3-Me, fumarate salt	33.7 ± 1.7	219 ± 42	210 ± 43

*ND, not determined*.

By virtue of its well-balanced *in vitro* activities towards the three targets, **5k** was selected for further investigation. According to the concept of MTDL and the synergy theoretically displayed by the association of activities into a sole compound, **5k** has been preferred to more potent compounds (e.g., **5m**) but with unbalanced levels of activity towards the three targets. This derivative has then been used as its fumaric acid salt **10**. The *in vitro* activities of **10** are depicted in Table 3.

**Table 3:** (*h*)5-HT_6_R and (*h*)5-HT_4(a)_R pharmacological profile of compounds **2**, **9** and **10**.

	(*h*)5-HT_6_R			(*h*)5-HT_4(a)_R		
	Log(IC_50_)	% control antagonist response	Profile	Log(EC_50_)	% control agonist response	Profile
**2**	−6.2 ± 0.2 *n* = 2	101.2 ± 3.2 *n* = 2	Inverse agonist	-	-	-
**9**	-	-	-	−8.8 ± 0.2 *n* = 2	48.7 ± 5.2 *n* = 3*	Partial agonist
**10**	−5.0 ± 0.1 *n* = 2	93.4 ± 1.8 *n* = 2	Inverse agonist	−7.7 ± 0.0 *n* = 2	59.8 ± 2.5 *n* = 2	Partial agonist

SB271046 was used as a control 5-HT_6_R antagonist and 5-HT as control 5-HT_4_R agonist. *Result reported from Lecoutey et al. (2014).

**Figure 4:**
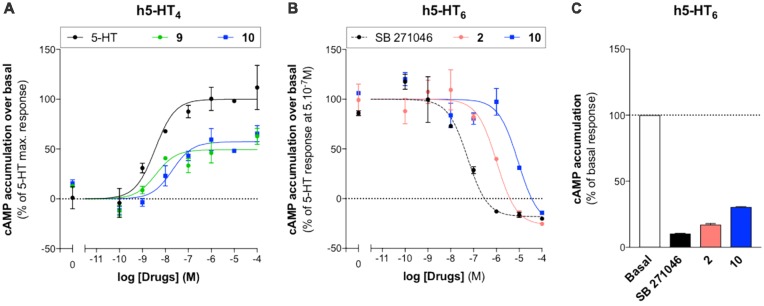
Pharmacological profile of compounds **2**, **9** and **10**. Representative experiments illustrating agonist activities of **9** and **10** towards (*h*)5-HT_4(a)_R **(A)** and antagonist activities of SB271046, **2** and **10** towards (*h*)5-HT_6_R stimulated with 5.10^−7^M of 5-HT [**B**; data are means ± standard error of the mean (SEM); *n* = 2]. **(C)** Inverse agonist activities of SB271046, **2** and **10** (10^−4^M) towards (*h*)5-HT_6_R in absence of 5-HT (means ± SEM of two independent experiments performed in duplicates).

The pharmacological profile of **10** was established towards (*h*)5-HT_4_R and (*h*)5-HT_6_R, respectively. It acts as a partial agonist towards (*h*)5-HT_4_R, in a similar manner as RS67333 (**9**), and as an inverse agonist towards (*h*)5-HT_6_R, in a similar manner as SB271046, used as a control 5-HT_6_R antagonist, and idalopirdine (**2**; Figure 4 and Table 3). Indeed, in our hands, these three compounds decreased cAMP production below the basal, in the absence of agonist (Figure 4C).

### *In vivo* Results

Whatever the dose tested (1, 10 and 100 mg/kg), preliminary toxicological and pharmacological screening of **10** (Table 4) did not show any deleterious signs, suggesting thus a LD_50_ quite higher than 100 mg/kg.

**Table 4:** Pharmacological and toxicological properties of **10**.

Compound	Doses (mg/kg)	LD_50_ (mg/kg)	Symptoms (subtoxic doses)
**10**	1–10–100	>100	No symptoms
Amphetamine	2		Hyperactivity
			Exopthalmy
			Irritability
Chlorpromazine	10		Hypoactivity
			Ataxia
			Sleep

**Figure 5:**
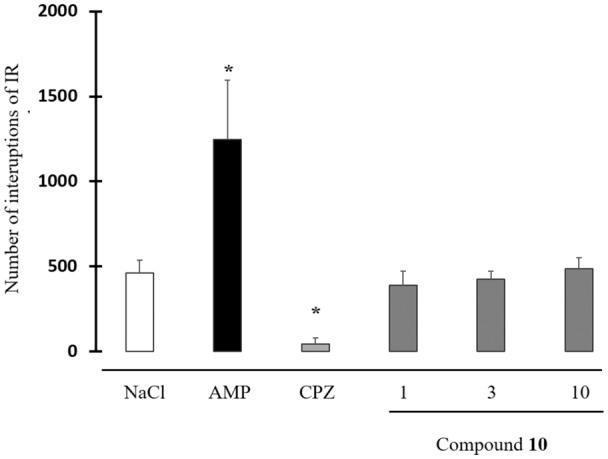
Effect of compound **10** on spontaneous locomotor activity. Data are expressed as the mean ± standard deviation (*n* = 8). Drugs were administered intraperitoneally (IP) 30 min before the behavioral test. Compound **10**: 1–3–10 mg/kg; AMP: amphetamine 2 mg/kg; CPZ: chlorpromazine 3 mg/kg [*p < 0.05 NaCl, Student-Newman-Keuls (SNK) test].

None of the tested doses of **10** (1, 3 and 10 mg/kg) did neither modify spontaneous locomotor activity (Figure 5).

**Figure 6:**
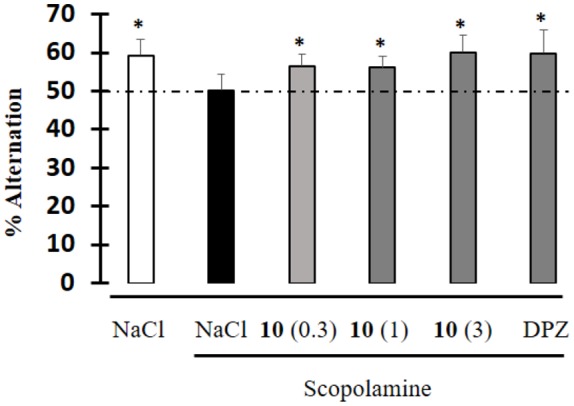
Effect of compound **10** on scopolamine-induced impairment during the spontaneous alternation test. Data are expressed as the mean ± standard deviation (*n* = 8). Drugs were administered IP 30 min before the test, and scopolamine was administered SC 20 min before the test. Compound **10**: 0.3, 1, 3 mg/kg; DPZ: Donepezil; 1 mg/kg; Scopolamine: 0.5 mg/kg (*p < 0.05 vs. 50%; univariate *t*-test).

Moreover, an anti-amnesic effect was observed in the scopolamine-induced spontaneous alternation deficit test, after IP administration of **10** at doses starting from 0.3 mg/kg (Figure 6). The degree of effect was similar to that of the dual-targeted compound donecopride in the same dose range (Rochais et al., 2015).

## Discussion

Trying to establish SAR for MTDL is always quite challenging taking into consideration that the structural requirements can be divergent between the expected interactions with the different targets. Considering the three present targets, the SAR appeared similar between AChE and 5-HT_4_R, but partially different from those observed for 5-HT_6_R.

Indeed, with regard to AChE and 5-HT_4_R activities, the best substituent of the piperidine moiety of compounds **5** is the unsubstituted benzyl group (**5a**). According to our previous work (Yahiaoui et al., 2016), the nature of the substitution of a phenyl group distant from the piperidine moiety could influence in a great manner the affinity of the ligands towards both 5-HT receptors. For the two targets, poly or bulky substituents (Br, I, OBn, OMe, NO_2_), appear detrimental to the activities (**5c, g, i, l, n**) while a smaller one (OH, NH_2_, F, Me) seems likely to relatively maintain the activity (**5h, p, m, d, j, o**), especially when present in para position (**5p**).

Conversely, the 5-HT_6_R affinity appears linked to the substitution of the benzyl group within the studied scaffold, since the unsubstituted derivative **5a** was devoid of such an activity. Further, small substituents (OH, F) do not favor, this time, the affinity (**5d, h, j, o, p**), whatever their position on the phenyl ring, while bulkier ones (Cl, Br, I, Me, OMe) promote it, especially in *ortho* or *meta* positions (**5b, c, e, f, i, k, l**).

These contradictory requirements explain that **5k** with a methyl group in *meta* position displayed balanced activities towards the three targets and was chosen for further investigation under its fumarate form (**10**). Indeed, according to the MTDL concept (Cavalli et al., 2008), pleiotropic ligand could present moderate *in vitro* activities toward their targets that could, however, lead to potent and promising *in vivo* activities explained by their synergistic effect (Prati et al., 2015).

Of interest, compound **10**, idalopirdine and SB271046 were able to reverse the constitutive activity of the 5-HT_6_ receptor, thus demonstrating an inverse agonist profile. Such a property is of high interest regarding this receptor as it is known to possess a high constitutive activity in the brain, exerting a tonic activity (Duhr et al., 2014; Deraredj Nadim et al., 2016). Regarding the 5-HT_4_R, **10** demonstrated a partial agonist activity similar to the one of **9**, which had proven its efficiency to prevent amyloid production in a mouse model of AD (Hashimoto et al., 2012; Giannoni et al., 2013; Baranger et al., 2017).

The main result of the behavioral study concerns the anti-amnesic effect of **10** in a model of scopolamine-induced deficit of working memory from the dose of 0.3 mg/kg.

Although no pharmacokinetic data are available for compound **10** yet, this result indicates that it reaches the general circulation and crosses the blood-brain barrier upon IP administration. The anti-amnesic effect of compound **10** suggests that the co-modulation of the three targets could be a therapeutic strategy of interest in the field of memory impairments. The anti-amnesic properties of the compound **10**, together with the absence of any deleterious signs during the pharmacological screening (even when tested at high dose: 100 mg/kg), are arguing in favor of **10** as a new promising drug, with excellent general tolerance.

## Conclusion

We have synthesized a variety of compounds possessing both *in vitro* and *in vivo* activities towards three therapeutic targets (5-HT_4_R/5-HT_6_R, AChE) in the potential treatment of AD. The use of these innovative MTDL which may exhibit a greater benefit than single-targeted drugs towards the neuropathological hallmarks of the disease and the associated behavioral deficits will require further studies in animal models and potential future clinical trials to demonstrate their efficacy.

## Associated Content

Analytical data of final compounds. This material is available free of charge *via* the Internet at http://pubs.acs.org.

## Data Availability

All datasets generated for this study are included in the manuscript and/or the Supplementary Files.

## Ethics Statement

All experiments were conducted in agreement with the European Directives and French law on animal experimentation (personal authorization n° 14-17 for MB and 14-60 for TF).

## Author Contributions

The manuscript was written through contributions of SC, TF, MB, CR and PD. All authors have given approval to the final version of the manuscript.

## Conflict of Interest Statement

The authors declare that the research was conducted in the absence of any commercial or financial relationships that could be construed as a potential conflict of interest.

## Abbreviations

AD: Alzheimer’s disease
ACh: acetylcholine
AChE: acetylcholinesterase
AChEI: acetylcholinesterase inhibitor
DPZ: donepezil
MTDL: multi-target directed ligands.
