# Supplementary material for: A Novel in vivo Anti-amnesic Agent, Specially Designed to Express Both Acetylcholinesterase (AChE) Inhibitory, Serotonergic Subtype 4 Receptor (5-HT4R) Agonist and Serotonergic Subtype 6 Receptor (5-HT6R) Inverse Agonist Activities, With a Potential Interest Against Alzheimer’s Disease
Source: Front Aging Neurosci. 2019 Jun 19;11:148. doi: 10.3389/fnagi.2019.00148 (PMC6611404; doi:10.3389/fnagi.2019.00148)

Supporting Information

A novel *in vivo* antiamnesic agent, specially designed to express both acetylcholinesterase (AChE) inhibitory, serotonergic subtype 4 receptor (5-HT$_4$R) agonist and serotonergic subtype 6 receptor (5-HT$_6$R) inverse agonist activities, with a potential interest against Alzheimer's disease.

Bérénice Hatat,[1,2‡] Samir Yahiaoui,[1‡] Cédric Lecoutey,[1] Audrey Davis,[1] Thomas Freret,[3] Michel Boulouard,[3] Sylvie Claeysen,[2] Christophe Rochais,[1*] Patrick Dallemagne[1*]

[1] Normandie Univ, UNICAEN, Centre d'Etudes et de Recherche sur le Médicament de Normandie (CERMN), Caen, France

[2] IGF, Univ. Montpellier, CNRS, INSERM, Montpellier, France

[3] Normandie Univ, UNICAEN, INSERM, U1075, COMETE, Caen, France

LC-MS analyses

The purities of all tested compounds were analyzed by LC−MS, with the purity all being higher than 95%. Analyses were performed using a Waters Alliance 2695 as separating module (column XBridge C18 2.5 μM/4.6x50 mM) using the following gradients: A (95%)/B (5%) to A (5%)/B (95%) in 4.00 min. This ratio was hold during 1.50 min before return to initial conditions in 0.50 min. Initial conditions were then maintained for 2.00 min (A = H_2O, B = CH_3CN; each containing HCOOH: 0.1%). MS were obtained on a SQ detector by positive ESI.

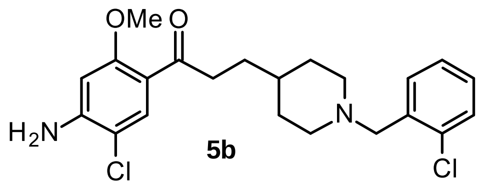
OMe
O
H2N
Cl
5b
N
Cl

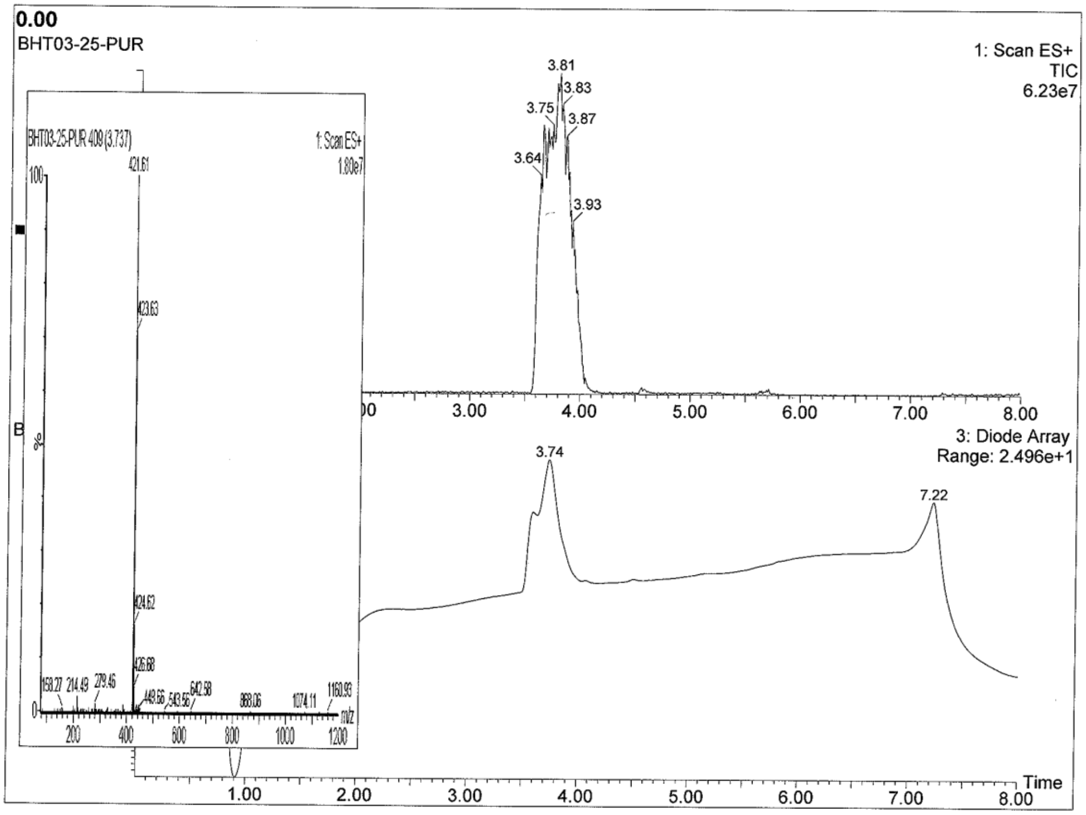
0.00
BHT03-25-PUR
1: Scan ES+
TIC
6.23e7
3.81
3.83
3.75
3.87
3.64
3.93
3: Diode Array
Range: 2.496e+1
3.74
7.22
Time
BHT03-25-PUR 409 (3.737)
1: Scan ES+
1.80e7
421.61
423.63
424.62
426.68
%
158.27
214.49
279.46
448.66
543.56
642.58
868.06
1074.11
1160.93
m/z

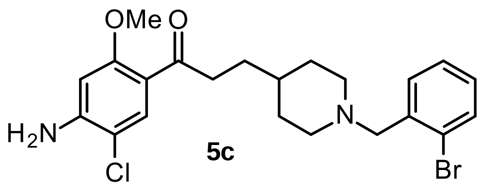

OMe
O
H2N
Cl
5c
N
Br

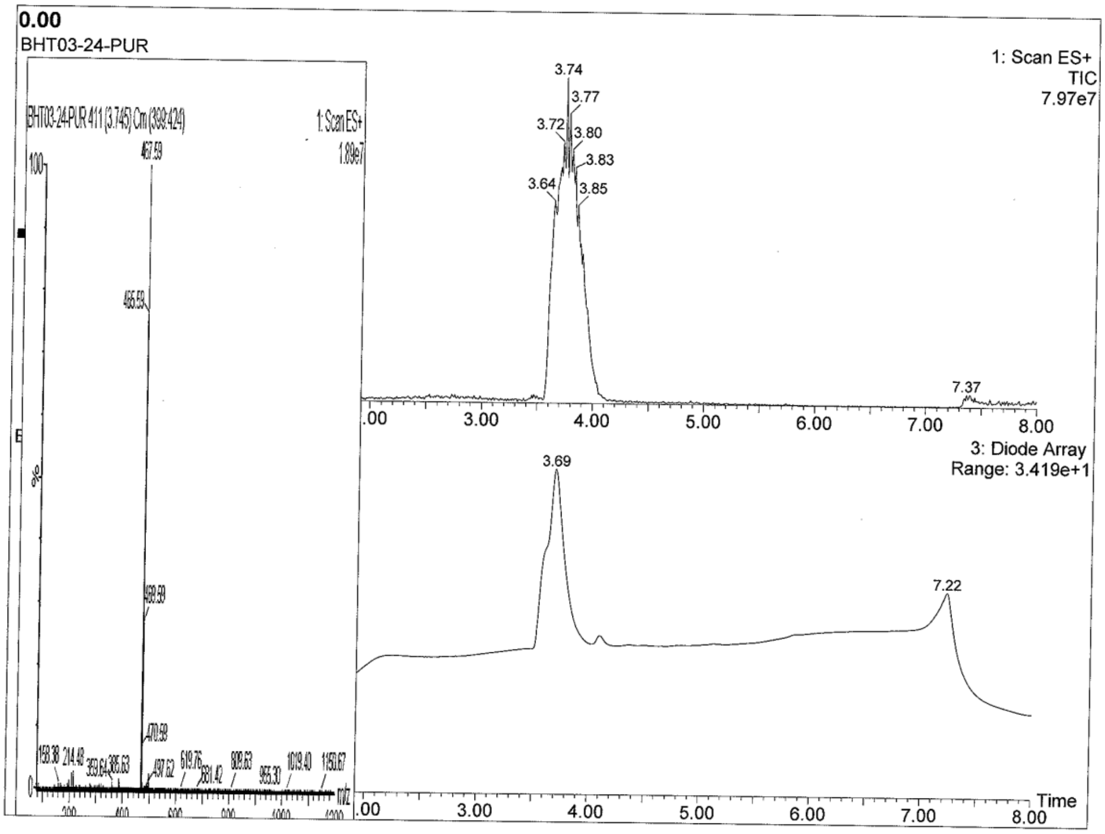

0.00
BHT03-24-PUR
1: Scan ES+
TIC
7.97e7
3: Diode Array
Range: 3.419e+1
Time

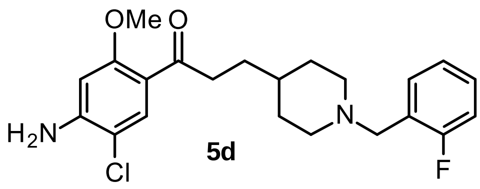
OMe
O
H2N
Cl
N
F

5d

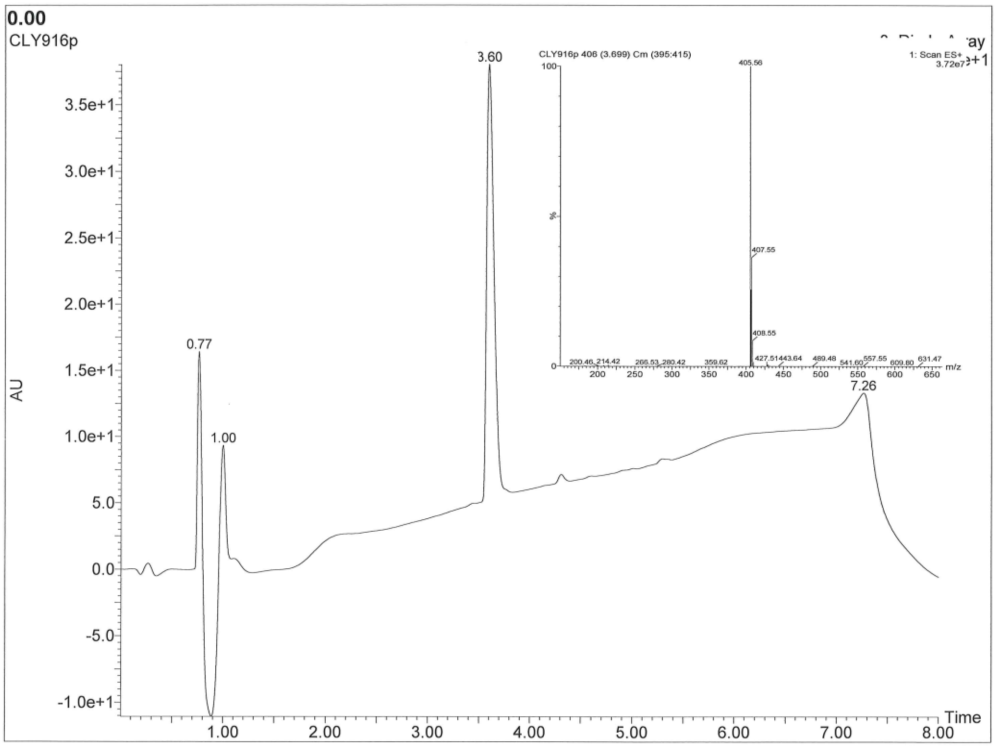
0.00
CLY916p
AU
Time
3.60
0.77
1.00
7.26
CLY916p 406 (3.699) Cm (395:415)
1: Scan ES+
3.72e7
405.56
407.55
408.55
m/z

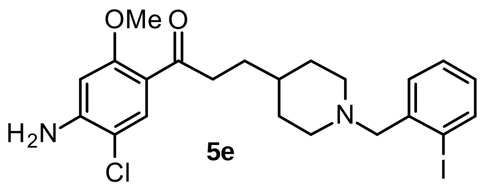
OMe
O
H2N
Cl
N
I

5e

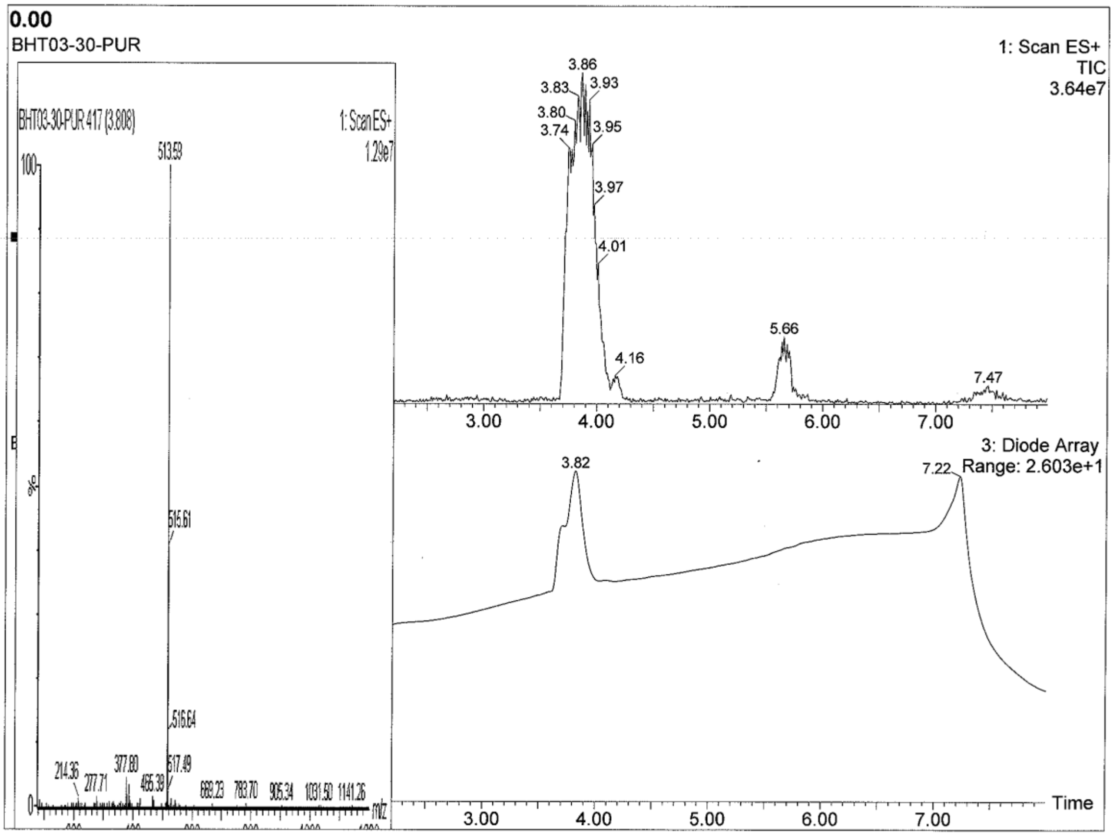
0.00
BHT03-30-PUR
BHT03-30-PUR 417 (3.808)
1: Scan ES+
1.29e7
513.59
100
%
515.61
516.64
517.49
214.36
277.71
377.60
465.38
668.23
783.70
905.34
1031.50
1141.26
m/z
1: Scan ES+
TIC
3.64e7
3.86
3.83
3.93
3.80
3.74
3.95
3.97
4.01
4.16
5.66
7.47
3.00
4.00
5.00
6.00
7.00
3: Diode Array
Range: 2.603e+1
3.82
7.22
Time

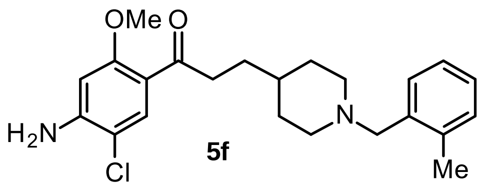
OMe
O
H2N
Cl
N
Me

5f

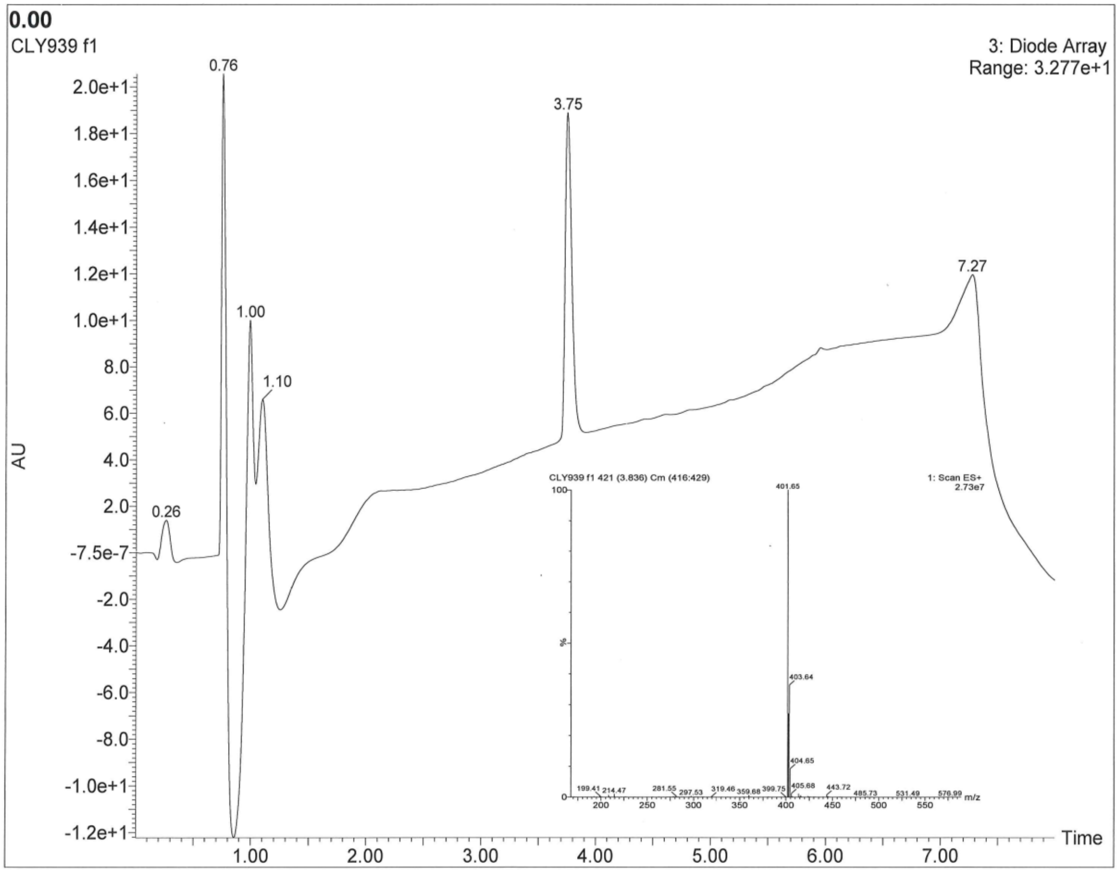
0.00
CLY939 f1
3: Diode Array
Range: 3.277e+1
AU
Time
0.26
0.76
1.00
1.10
3.75
7.27
CLY939 f1 421 (3.836) Cm (416:429)
1: Scan ES+
2.73e7
401.65
403.64
404.65
m/z

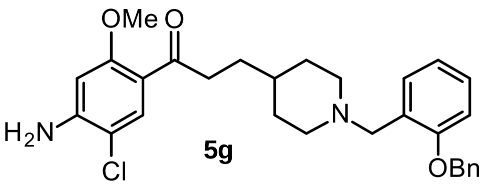

5g

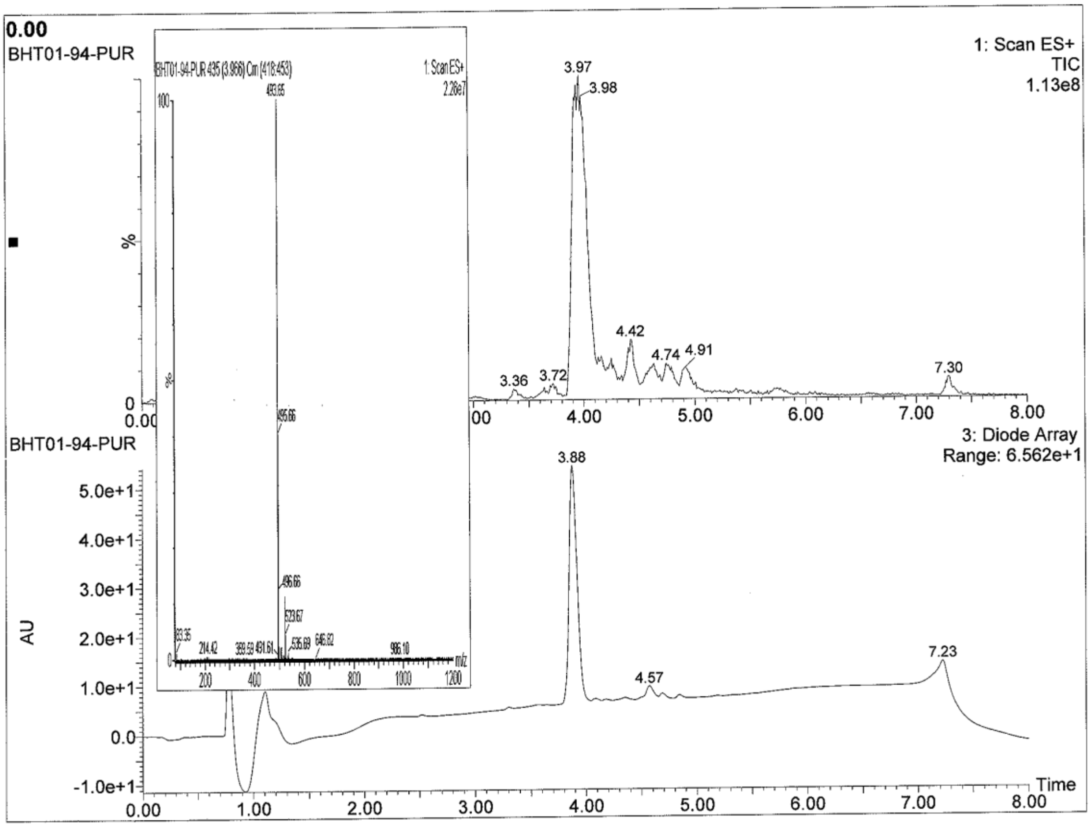

0.00
BHT01-94-PUR
1: Scan ES+
TIC
1.13e8
3.97
3.98
4.42
4.74
4.91
3.36
3.72
7.30
BHT01-94-PUR
3: Diode Array
Range: 6.562e+1
3.88
4.57
7.23
AU
Time
5.0e+1
4.0e+1
3.0e+1
2.0e+1
1.0e+1
0.0
-1.0e+1
0.00
1.00
2.00
3.00
4.00
5.00
6.00
7.00
8.00
1: Scan ES+
2.28e7
493.65
495.66
496.66
523.67
m/z

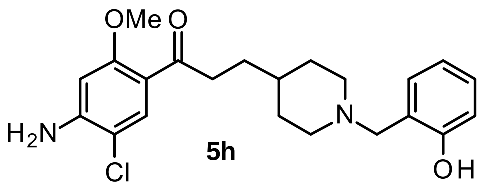

OMe
O
H2N
Cl
N
OH
5h

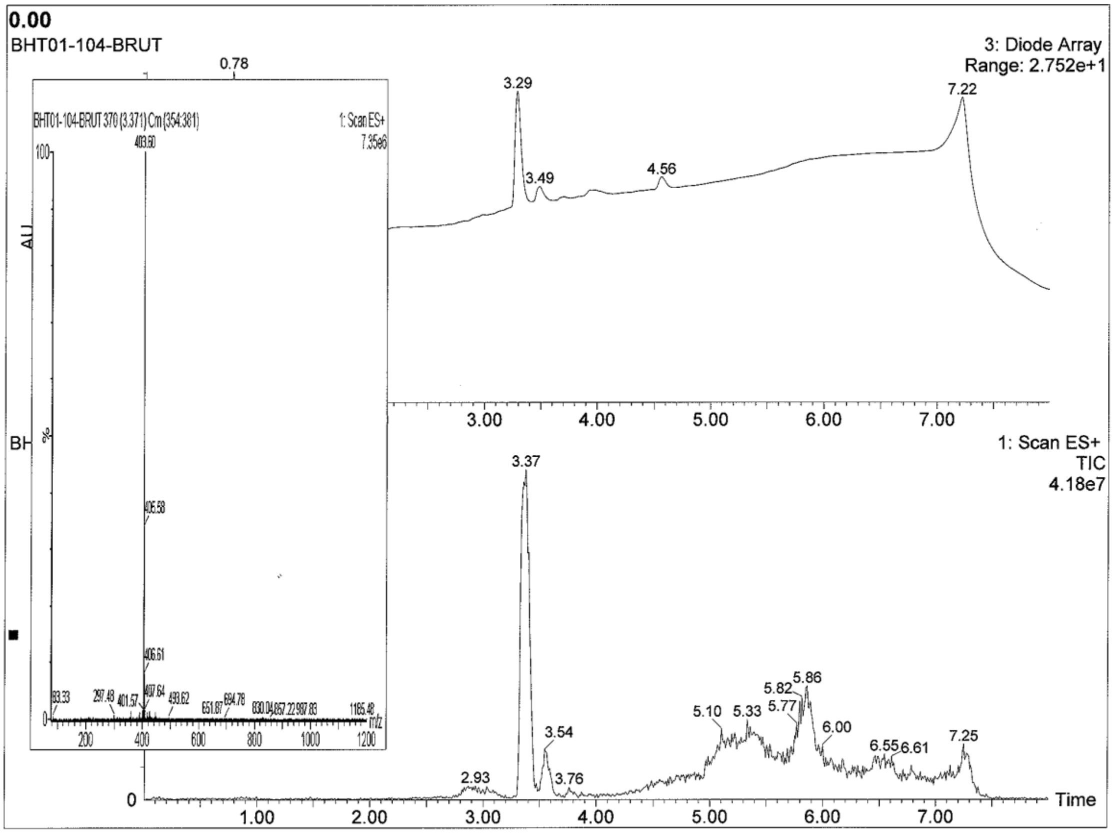

0.00
BHT01-104-BRUT
3: Diode Array
Range: 2.752e+1
3.29
3.49
4.56
7.22
0.78
BHT01-104-BRUT 370 (3.371) Cm (354:381)
1: Scan ES+
7.35e6
403.60
405.58
406.61
AU
1: Scan ES+
TIC
4.18e7
3.37
3.54
3.76
2.93
5.10
5.33
5.82
5.77
5.86
6.00
6.55
6.61
7.25
Time
1.00
2.00
3.00
4.00
5.00
6.00
7.00
m/z

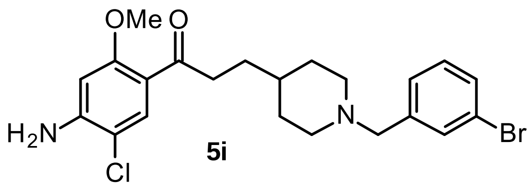
OMe
O
H2N
Cl
N
Br

5i

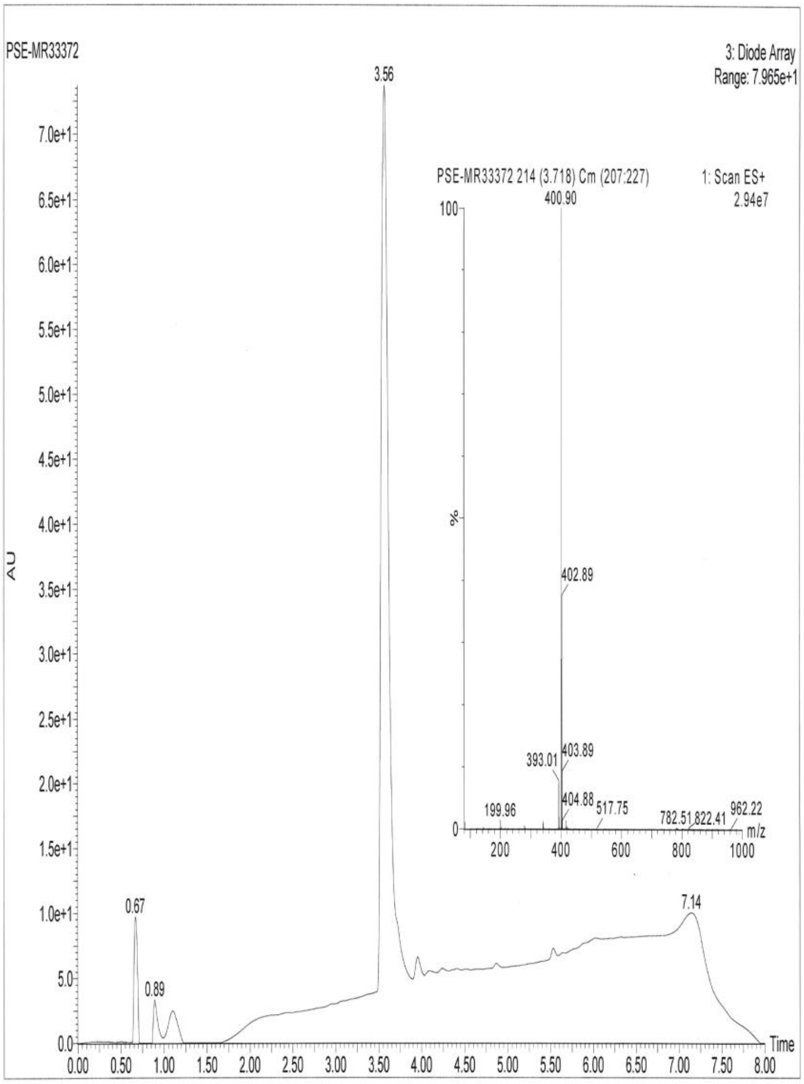
PSE-MR33372
3: Diode Array
Range: 7.965e+1
3.56
PSE-MR33372 214 (3.718) Cm (207:227)
1: Scan ES+
2.94e7
400.90
402.89
393.01
403.89
404.88
199.96
517.75
782.51
822.41
962.22
m/z
%
AU
0.67
0.89
7.14
Time

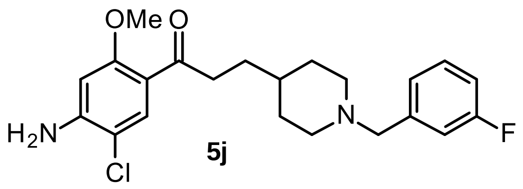
OMe
O
H2N
Cl
5j
N
F

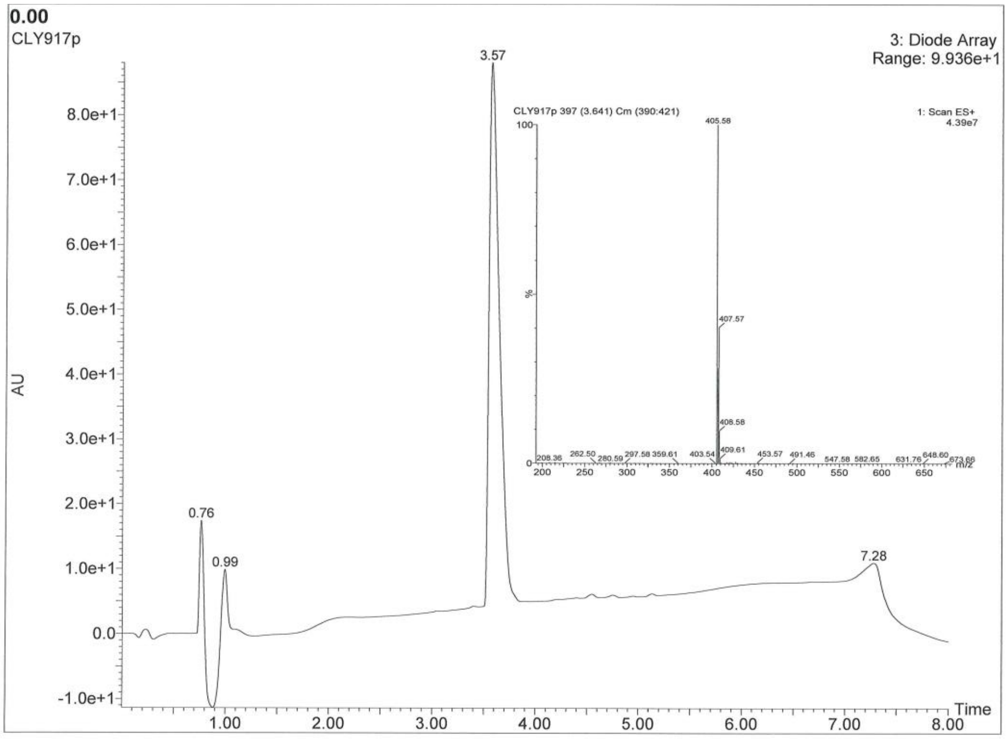
0.00
CLY917p
3: Diode Array
Range: 9.936e+1
AU
Time
3.57
0.76
0.99
7.28
CLY917p 397 (3.641) Cm (390:421)
1: Scan ES+
4.39e7
405.58
407.57
408.58
409.61
m/z

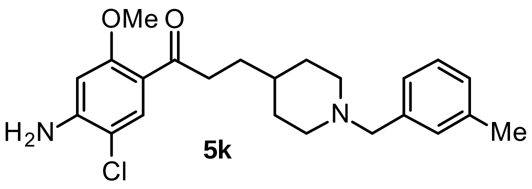
OMe
O
H_2N
Cl
N
Me

5k

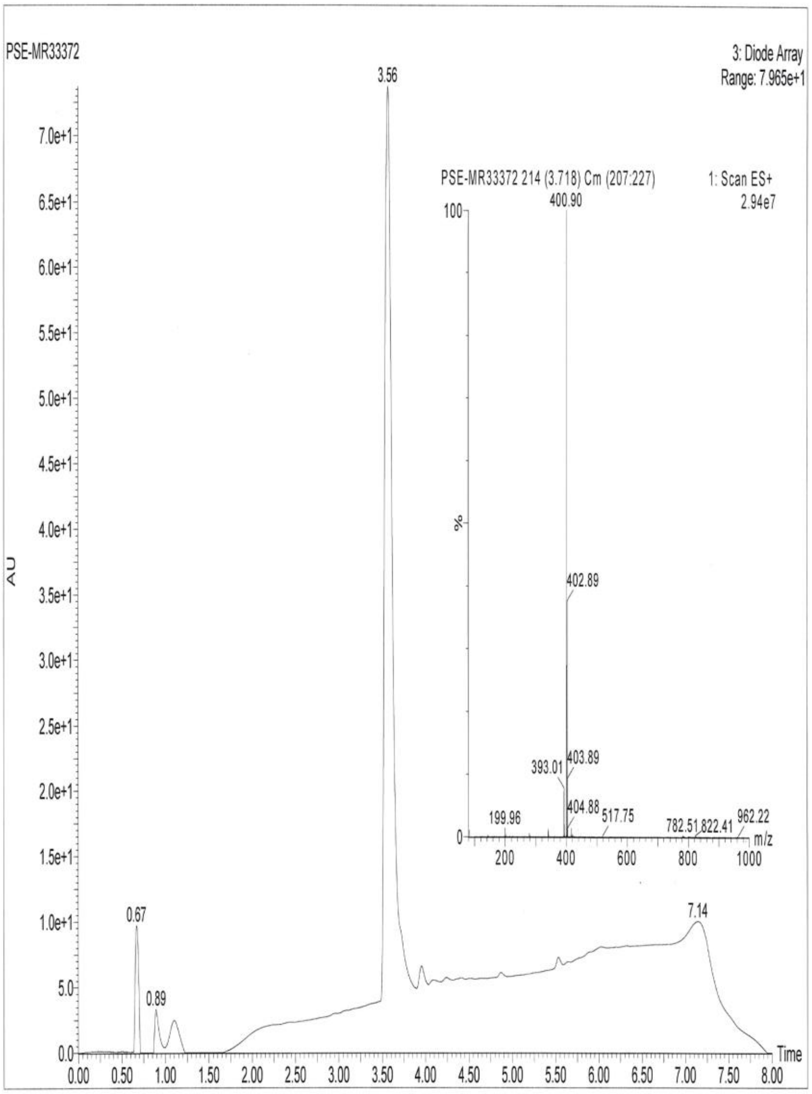
PSE-MR33372
3: Diode Array
Range: 7.965e+1
3.56
AU
7.0e+1
6.5e+1
6.0e+1
5.5e+1
5.0e+1
4.5e+1
4.0e+1
3.5e+1
3.0e+1
2.5e+1
2.0e+1
1.5e+1
1.0e+1
5.0
0.0
0.67
0.89
7.14
Time
0.00 0.50 1.00 1.50 2.00 2.50 3.00 3.50 4.00 4.50 5.00 5.50 6.00 6.50 7.00 7.50 8.00
PSE-MR33372 214 (3.718) Cm (207:227)
1: Scan ES+
2.94e7
100
400.90
%
402.89
393.01
403.89
199.96
404.88
517.75
782.51
822.41
962.22
0
m/z
200 400 600 800 1000

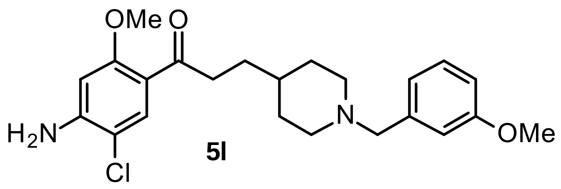

5l

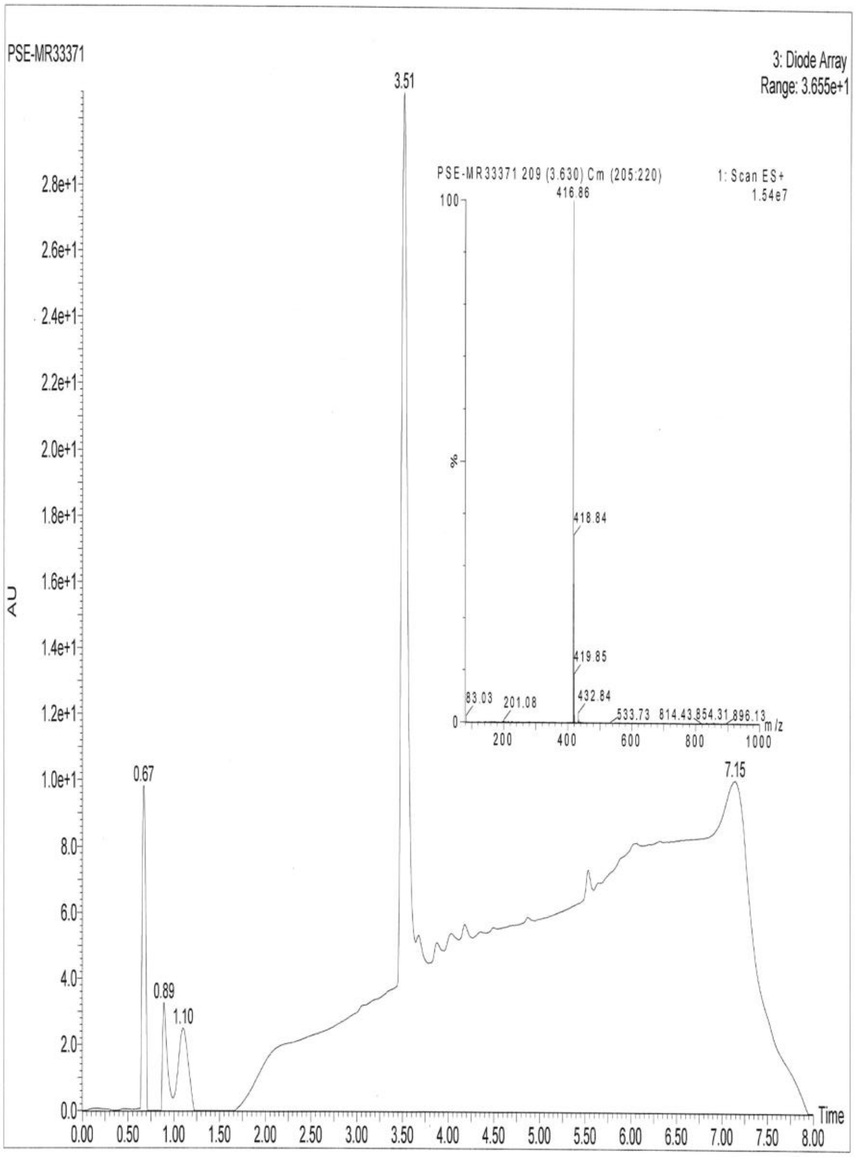

PSE-MR33371
3: Diode Array
Range: 3.655e+1
3.51
PSE-MR33371 209 (3.630) Cm (205:220)
1: Scan ES+
1.54e7
416.86
418.84
419.85
432.84
83.03
201.08
533.73
814.43
854.31
896.13
m/z
%
AU
0.67
0.89
1.10
7.15
Time

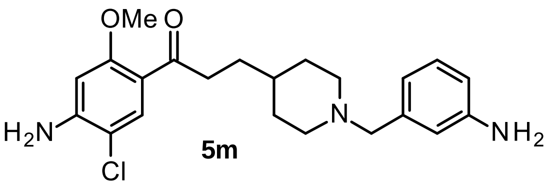

5m

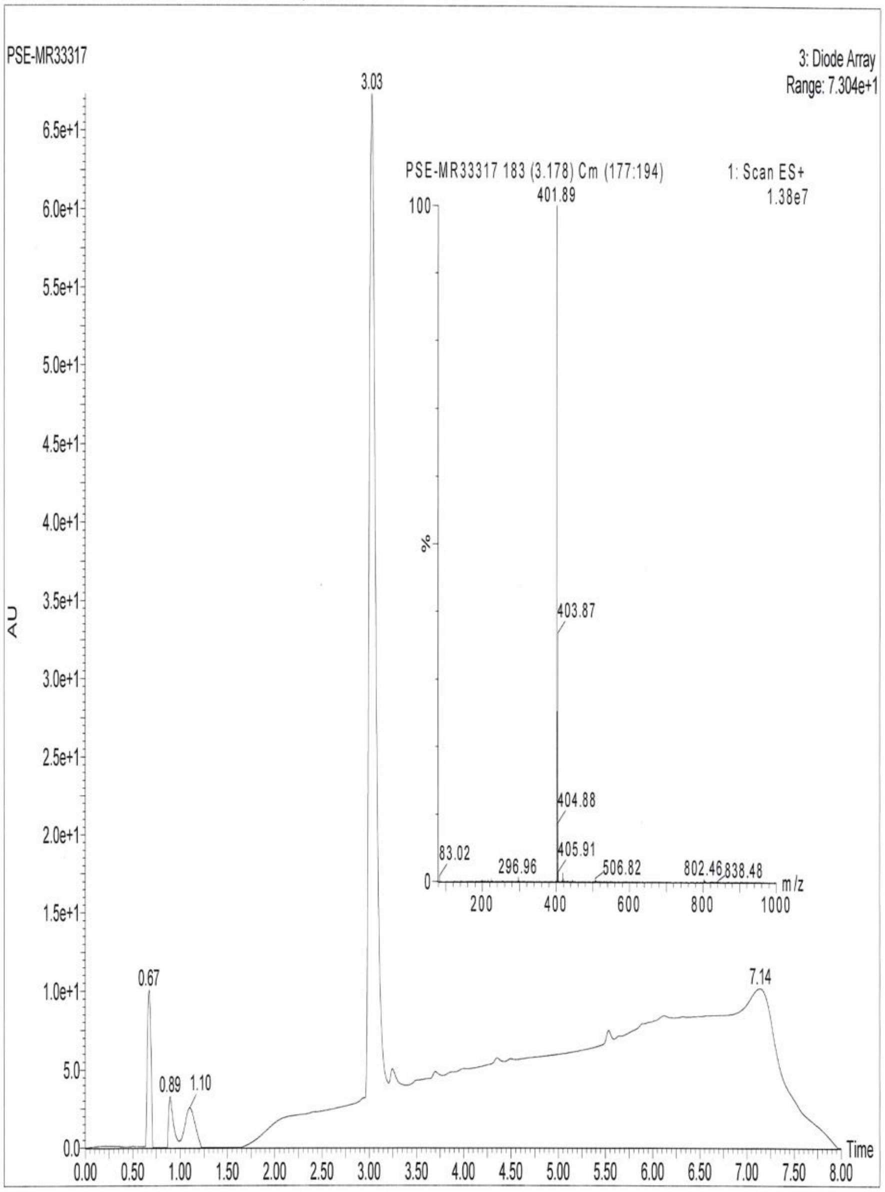
PSE-MR33317
3: Diode Array
Range: 7.304e+1
3.03
PSE-MR33317 183 (3.178) Cm (177:194)
1: Scan ES+
1.38e7
401.89
403.87
404.88
405.91
83.02
296.96
506.82
802.46
838.48
%
m/z
AU
0.67
0.89
1.10
7.14
Time

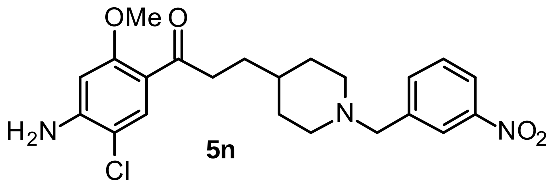

5n

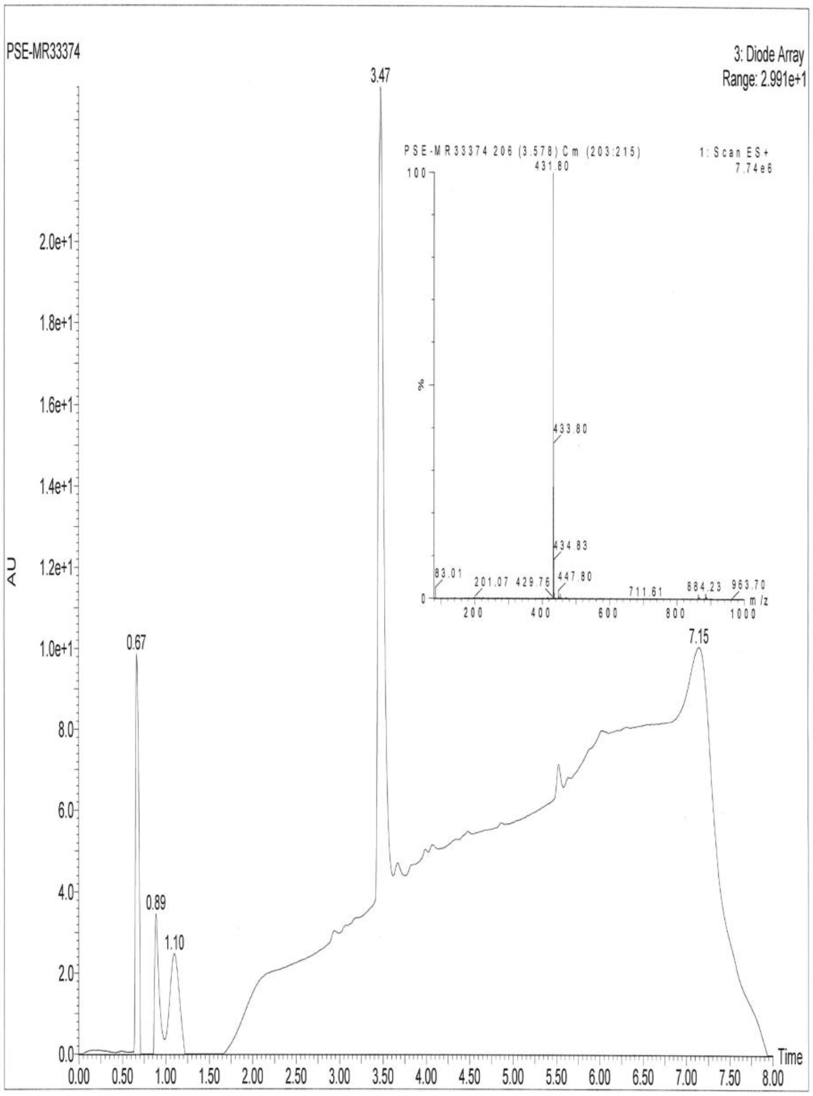

PSE-MR33374
3: Diode Array
Range: 2.991e+1
3.47
PSE-MR33374 206 (3.578) Cm (203:215)
1: Scan ES+
7.74e6
431.80
433.80
434.83
447.80
83.01
201.07
429.76
711.61
884.23
963.70
m/z
%
0.67
0.89
1.10
7.15
AU
Time

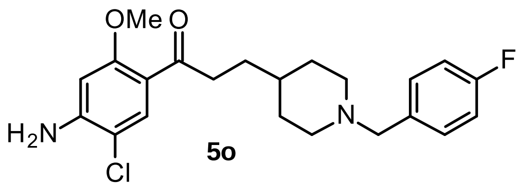
OMe
O
F
H_2N
N
Cl

5o

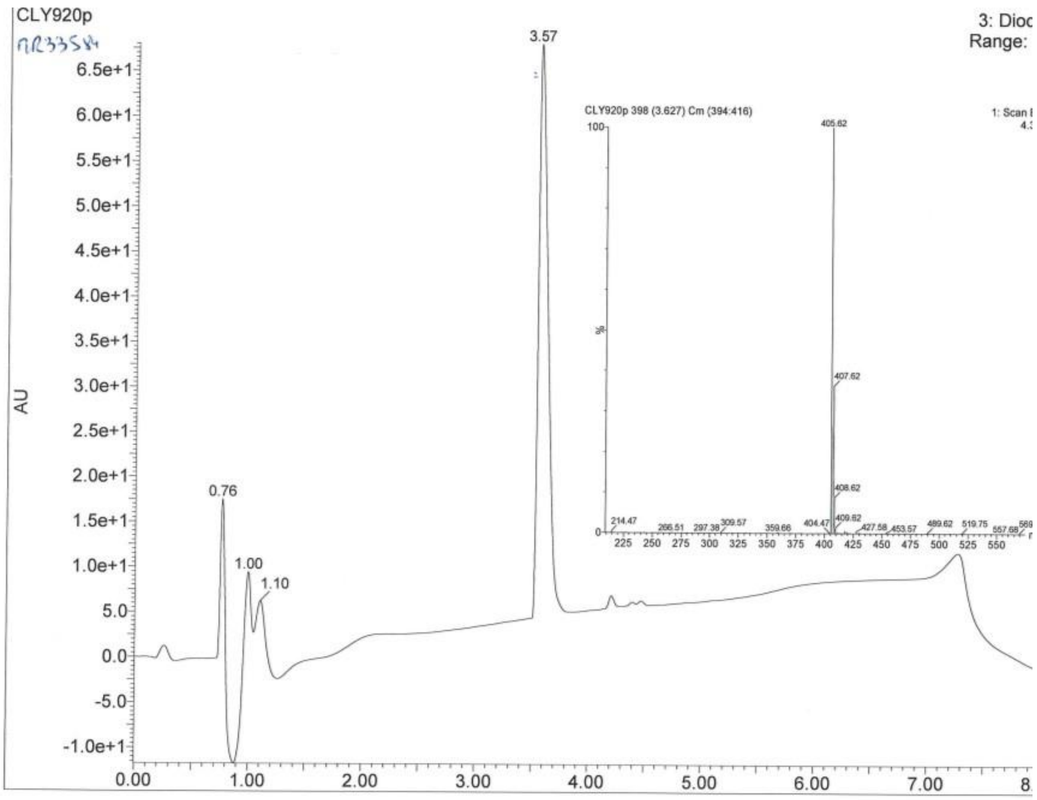
CLY920p
3: Dioc
Range:
AU
3.57
0.76
1.00
1.10
CLY920p 398 (3.627) Cm (394:416)
1: Scan
405.62
407.62
408.62
214.47
266.51
297.38
309.57
359.66
404.47
409.62
427.58
453.57
489.62
519.75
557.68

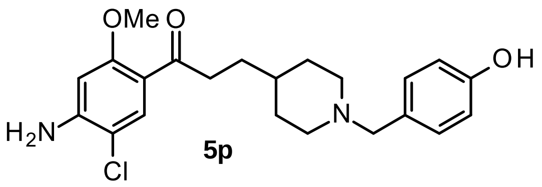
OMe
O
OH
H2N
N
Cl

5p

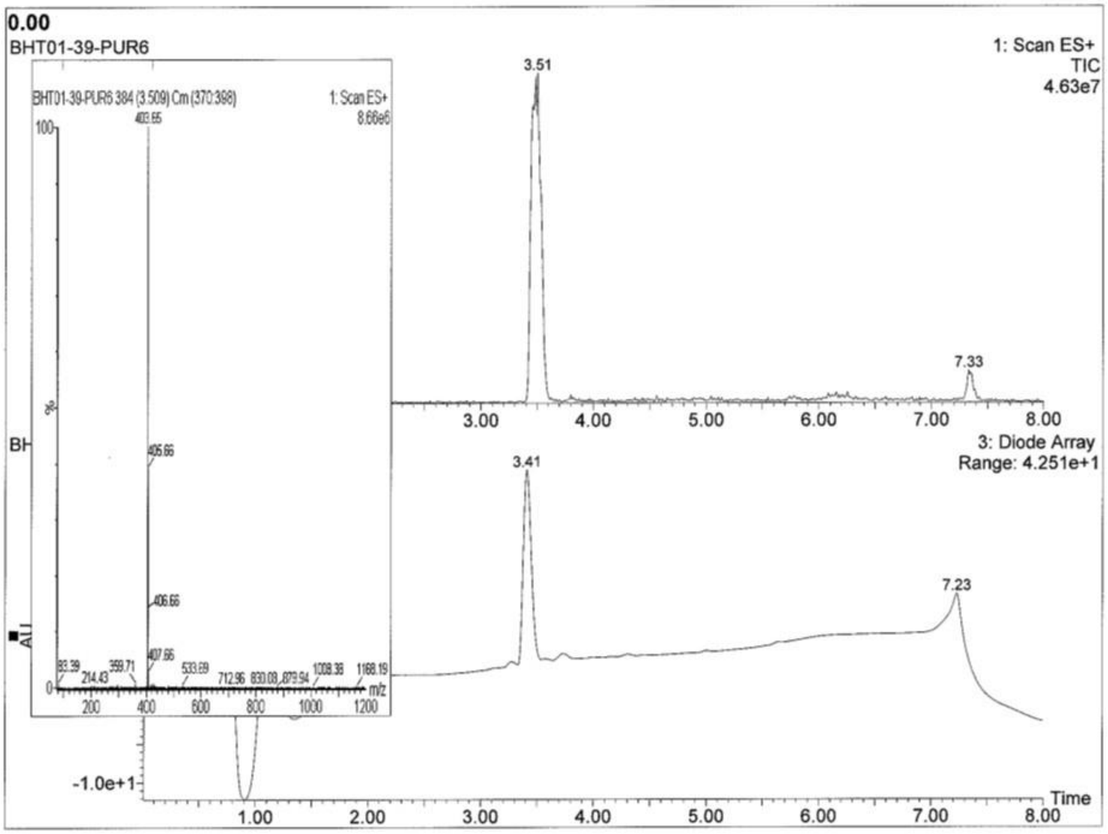
0.00
BHT01-39-PUR6
1: Scan ES+
TIC
4.63e7
3.51
7.33
3.00
4.00
5.00
6.00
7.00
8.00
3: Diode Array
Range: 4.251e+1
3.41
7.23
-1.0e+1
Time
1.00
2.00
BHT01-39-PUR6 384 (3.509) Cm (370:398)
1: Scan ES+
8.66e6
403.65
100
%
405.66
406.66
83.39
214.43
359.71
407.66
533.69
712.96
830.08
879.94
1008.38
1168.19
0
m/z
200
400
600
800
1000
1200

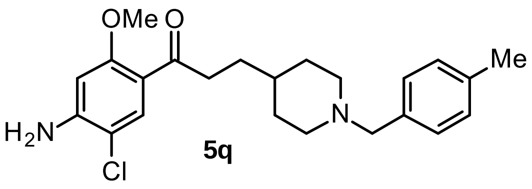
OMe
O
Me
N
H2N
Cl

5q

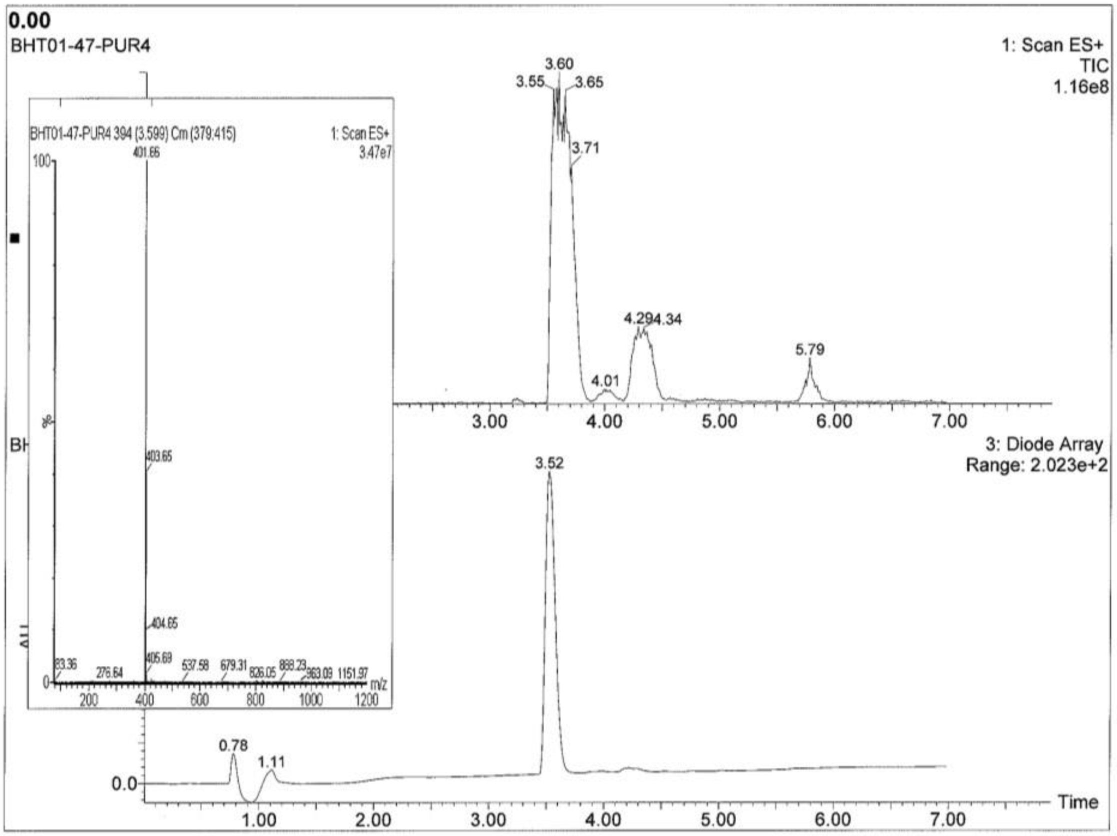
0.00
BHT01-47-PUR4
1: Scan ES+
TIC
1.16e8
3.55
3.60
3.65
3.71
4.01
4.29
4.34
5.79
3.00
4.00
5.00
6.00
7.00
3: Diode Array
Range: 2.023e+2
3.52
0.78
1.11
0.0
1.00
2.00
Time
BHT01-47-PUR4 394 (3.599) Cm (379:415)
1: Scan ES+
3.47e7
100
401.66
%
403.65
404.65
83.36
276.64
405.69
537.58
679.31
826.05
868.23
963.09
1151.97
0
200
400
600
800
1000
1200
m/z

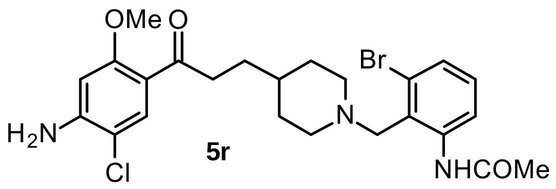
OMe
O
Br
H_2N
N
NHCOMe
Cl

5r

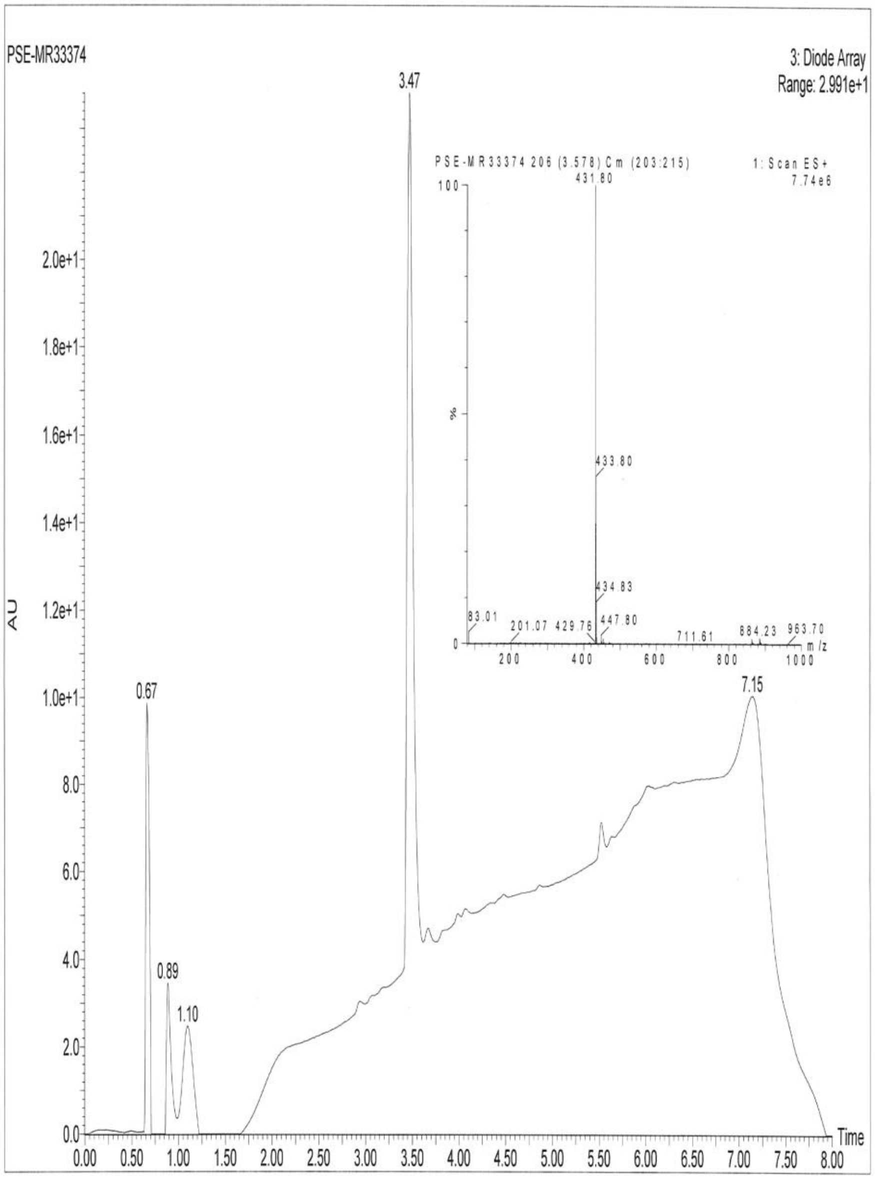
PSE-MR33374
3: Diode Array
Range: 2.991e+1
AU
Time
3.47
0.67
0.89
1.10
7.15
PSE-MR33374 206 (3.578) Cm (203:215)
1: Scan ES+
7.74e6
431.80
433.80
434.83
447.80
83.01
201.07
429.76
711.61
884.23
963.70
m/z
%

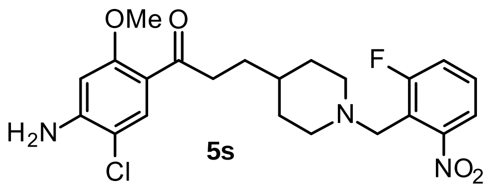

5s

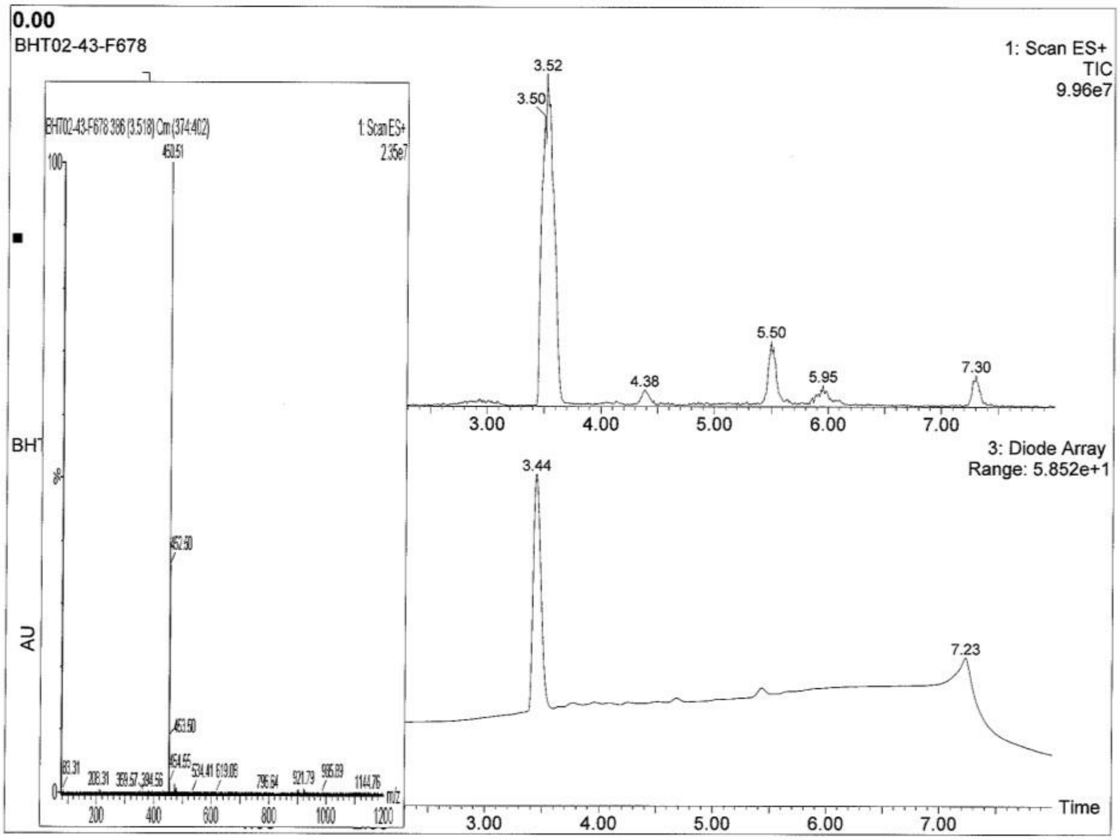
0.00
BHT02-43-F678
1: Scan ES+
TIC
9.96e7
3.52
3.50
4.38
5.50
5.95
7.30
3.00
4.00
5.00
6.00
7.00
3: Diode Array
Range: 5.852e+1
3.44
7.23
AU
Time
BHT02-43-F678 386 (3.518) Cm (374:402)
1: Scan ES+
2.35e7
450.51
452.50
453.50
m/z

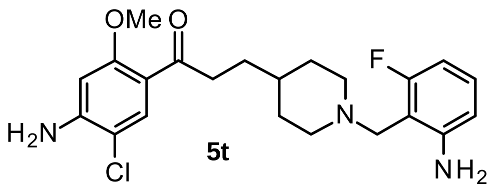
OMe
O
F
N
H2N
NH2
Cl

5t

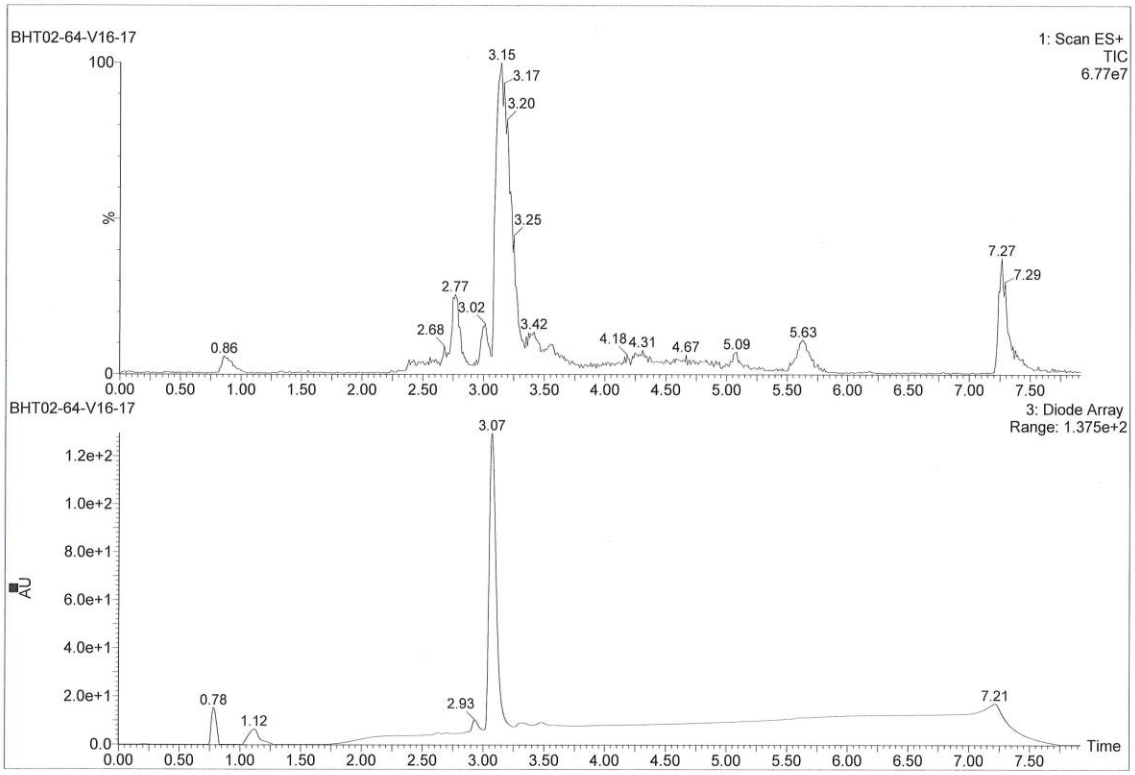
BHT02-64-V16-17
1: Scan ES+
TIC
6.77e7
100
%
0
3.15
3.17
3.20
3.25
2.77
3.02
2.68
3.42
0.86
4.18
4.31
4.67
5.09
5.63
7.27
7.29
0.00 0.50 1.00 1.50 2.00 2.50 3.00 3.50 4.00 4.50 5.00 5.50 6.00 6.50 7.00 7.50
BHT02-64-V16-17
3: Diode Array
Range: 1.375e+2
AU
1.2e+2
1.0e+2
8.0e+1
6.0e+1
4.0e+1
2.0e+1
0.0
3.07
0.78
1.12
2.93
7.21
Time
0.00 0.50 1.00 1.50 2.00 2.50 3.00 3.50 4.00 4.50 5.00 5.50 6.00 6.50 7.00 7.50

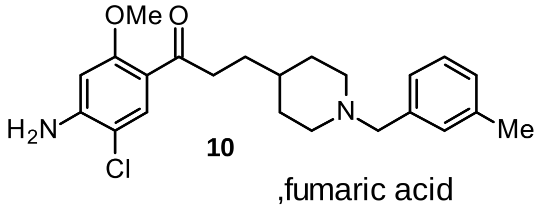

10

,fumaric acid

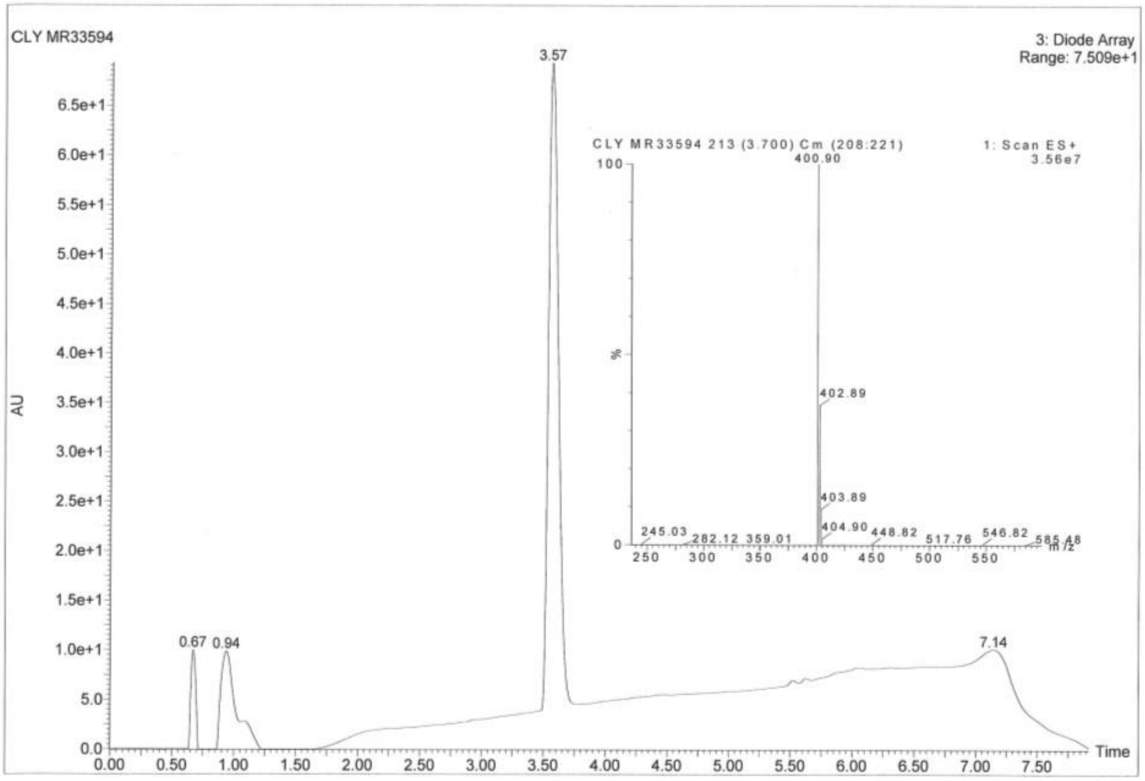

Supplement: Supplementary file 1 [file Data_Sheet_1.PDF]
